# Conformationally Confined Emissive Cationic Macrocycle with Photocontrolled Organelle‐Specific Translocation

**DOI:** 10.1002/advs.202201962

**Published:** 2022-06-17

**Authors:** Xiaoyun Dong, Xianyin Dai, Guorong Li, Ying‐Ming Zhang, Xiufang Xu, Yu Liu

**Affiliations:** ^1^ College of Chemistry State Key Laboratory of Elemento‐Organic Chemistry Nankai University Tianjin 300071 P. R. China; ^2^ Haihe Laboratory of Sustainable Chemical Transformations Tianjin 300192 P. R. China

**Keywords:** conformation confinement, macrocycle, singlet oxygen, supramolecular chemistry, targeted cell‐imaging

## Abstract

The optimization of molecular conformation and aggregation modes is of great significance in creation of new luminescent materials for biochemical research and medical diagnostics. Herein, a highly emissive macrocycle (**1**) is reported, which is constructed by the cyclization reaction of triphenylamine with benzyl bromide and exhibits very distinctive photophysical performance both in aqueous solution and the solid state. Structural analysis reveals that the **1** can form self‐interpenetrated complex and emit bright yellow fluorescence in the crystal lattice. The distorted yet symmetrical structure can endow **1** with unique two‐photon absorption property upon excitation by near‐infrared light. Also, **1** can be utilized as an efficient photosensitizer to produce singlet oxygen (^1^O_2_) both in inanimate milieu and under cellular environment. More intriguingly, due to the strong association of **1** with negatively charged biomacromolecules, organelle‐specific migration is achieved from lysosome to nucleus during the ^1^O_2_‐induced cell apoptosis process. To be envisaged, this conformationally confined cationic macrocycle with photocontrolled lysosome‐to‐nucleus translocation may provide a feasible approach for in situ identifying different biospecies and monitoring physiological events at subcellular level.

## Graphical abstract

A triphenylamine‐based tetracationic macrocycle not only exhibits distinctive photoluminescence behaviors both in solution and solid state via the conformation confinement effect but also undergoes a photocontrollable lysosome–nucleus translocation in the cell apoptosis process induced by the photosensitized singlet oxygen.

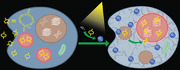

## 1 Introduction

The construction of well‐crafted artificial macrocyclic receptors occupies unique and important positions in the realm of supramolecular chemistry. Along with the rapid development in the past decades, diverse macrocyclic compounds have been created with defined topological structures and elaborate functions, which greatly expand the research scope for nanoscience and have inventive applications in material manufacturing.^[^ ^1^ ^]^ However, the commonly used representative macrocycles, including the parent crown ethers, cyclodextrins, and cucurbiturils, are spectroscopically transparent or possess rather weak absorption in the UV–vis region. These factors seriously hinder their development and applications, especially as photoluminescent materials in the biorelated fields. To alleviate these problems, some highly emissive macrocycles consisting of *π*‐conjugated components have drawn into the limelight because of their immense advantages in host–guest complexation and molecular recognition.^[^ ^2^ ^]^ It seems essential to construct new generation of macrocycles with superior photophysical performance and explore their functions in creation of biocompatible photoactive nanoarchitectures.^[^ ^3^ ^]^

Recently, supramolecular confinement has been emerged as a new concept and perspective to elucidate the photophysical origins of the obtained supramolecular nanoconstructs.^[^ ^4^ ^]^ On one hand, macrocyclic hosts inherently possess a 3D microenvironment, which can immobilize the conformation of heterogenous guests inside their cavities. This binding feature can induce the dramatic spectroscopic outcomes and photoluminescence enhancement of the encapsulated substrates.^[^ ^5^ ^]^ On the other hand, the supramolecular confinement can be also achieved by abundant intermolecular interactions among the homogeneous macrocycles, as exemplified by the aggregation‐induced emission of chromophores in the condensed states.^[^ ^6^ ^]^ The confined spaces arising from macrocyclic compounds provide bountiful opportunities for fabrication of bioactive photoluminescent materials, which cannot be simply realized by the acyclic counterparts in open chains.^[^ ^7^ ^]^

**Scheme 1:**
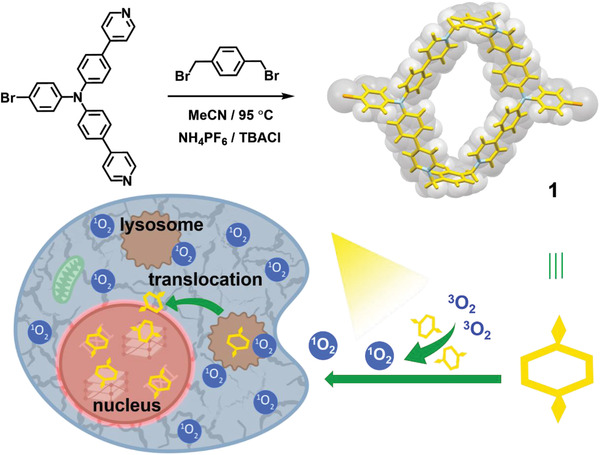
Synthesis of macrocycle 1 and its photocontrolled translocation from lysosome to nucleus upon ^1^O_2_ generation.

Inspired by the cyclization effect on molecular conformation and concomitant physicochemical properties, in this work, a triphenylamine‐based tetracationic macrocycle (**1**) is synthesized and its crystal structure is obtained by use of a slow evaporation method (**Scheme** **1**). Crystallographic analyses demonstrate that the crystal‐state structure of **1** adopts a pseudopolyrotaxane‐style arrangement, which is stabilized by the strong intermolecular *π*‐stacking interactions. Meanwhile, the macrocycle **1** possesses good photosensitizing ability of singlet oxygen (^1^O_2_) production and desired two‐photon absorption property upon excitation at 880 nm. More interestingly, when incubated in the cell milieu, **1** can directly migrate from lysosome to nucleus under the light irradiation (Movie S1, Supporting Information). Also, due to the high affinity toward nucleic acids with highly ordered structures, **1** gives greater phototoxicity toward cancer cells than the acyclic reference. It can be anticipated that our emissive macrocycle featuring the photocontrolled lysosome‐to‐nucleus translocation may hold great promises in organelle identification, cell‐fate monitoring, and even prodrug formulation.

## 2 Results and Discussion

**Figure 1:**
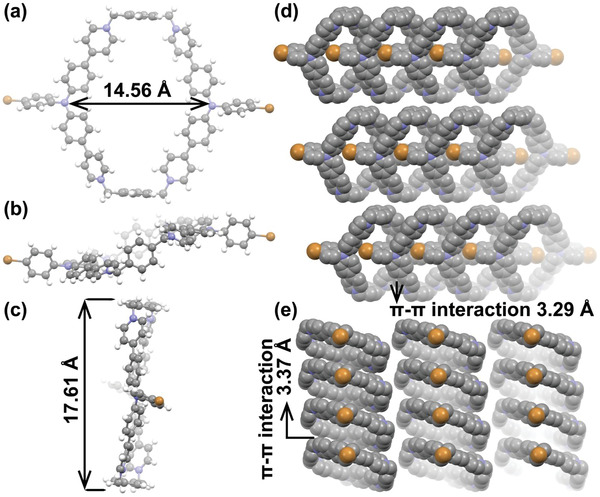
X‐ray crystal structures of **1∙4PF_6_** ^−^: a–c) individual units in different dimensions. Space‐fill structures shown along d) *a* and e) *b* axis, respectively.

The triphenylamine‐based tetracationic macrocycle **1** was conveniently synthesized via the cyclization reaction between pyridine‐conjugated triphenylamine and bis(bromomethyl)‐benzene under dilution condition (Chart S1 and Figures S1–S3, Supporting Information). The hexafluorophosphate (**1∙4PF_6_** ^−^) could be converted to the corresponding chloride salt (**1∙4Cl** ^−^) and increased the water solubility up to 50 × 10^−6^ m by counterion exchange, which is sufficient for the application in biosystems. In addition, the acyclic compound **2** was obtained in a similar way and used as the reference (Chart S2 and Figures S4 and S5, Supporting Information). The detailed synthetic routs are shown in the Supporting Information. Fortunately, the single crystal of **1∙4PF_6_** ^−^ was obtained as by slow evaporation of a water/acetonitrile mixed solution (1 × 10^−3^ m) at room temperature for 4 d. As shown in **Figure** **1**a–c, the individual unit of **1∙4PF_6_** ^−^ possesses a large‐sized hexahedral and electron‐deficient cavity with the height and width of 14.56 and 17.61 Å, respectively (Figure 1a–c). Meanwhile, the cavity of **1∙4PF_6_** ^−^ is distorted in shape. As result, the host–guest complexes between **1** and some known *π*‐aromatic planar substrates, including pyrene, porphyrin, and perylene, cannot be efficiently formed by the mutual *π*‐stacking interaction. Under such circumstance, it is believed that electrostatic attraction arising from four pyridinium sites in **1** may turn out to be a dominant role upon binding of some negatively charged substrates, especially in aqueous solution.^[^ ^8^ ^]^

After scrutinizing the crystallographic results, it is found that there are extensive intermolecular *π*‐stacking interactions in the spacing‐fill structure of **1∙4PF_6_** ^−^ and the centroid‐centroid distances were measured as 3.29 and 3.37 Å along *a* and *b* axles, respectively (Figure 1d,e). It is also shown that the bromophenyl group of **1∙4PF_6_** ^−^ intermolecularly penetrates into the cavity of neighboring macrocycle straight along *a* axle to form orderly pseudopolyrotaxanes (Figure 1d). In our case, the packing structures of **1∙4PF_6_** ^−^ are costabilized by the intermolecular *π*‐stacking interaction among the aromatic moieties and the mutual coordination with the counterions (Figure S6, Supporting Information). Apparently, theses multiple interactions in the crystal lattice would dramatically immobilize the whole molecular conformation of **1** and thus improve its photophysical outcomes in the solid state, as described below.

To further explore the photoluminescence behaviors, spectroscopic experiments were conducted in water and organic solution. As can be seen in Figure S7 in the Supporting Information, **1∙4PF_6_** ^−^ and its water‐soluble counterpart **1∙4Cl** ^−^ showed broad UV–vis absorption in the region of 350–520 nm. Meanwhile, compared to the maximum absorption peak of **1∙4PF_6_** ^−^ in acetonitrile, the one of **1∙4Cl** ^−^ gave large hypochromatic shift of 9 nm in water. Moreover, **1∙4PF_6_** ^−^ is weakly fluorescent in acetonitrile nm but gave very strong fluorescence emission at 590 nm in the solid state (Figures S8 and S9, Supporting Information). It is also noteworthy that **1∙4Cl** ^−^ could emit bright yellow light centered at 556 nm when excited at 440 nm (Figure S8, Supporting Information). Accordingly, the quantum yields of **1∙4Cl** ^−^ and **1∙4PF_6_** ^−^ were obtained as 13.7% in water and 17.5% in the solid state, respectively (Figures S10 and S11, Supporting Information). Subsequently, the fluorescent property of **1** was further studied in the mixed solvents. As depicted in Figure S12 in the Supporting Information, **1∙4Cl** ^−^ shows weak emission in pure dimethyl sulfoxide (DMSO) and *N*,*N*’‐dimethylformamide (DMF). Since **1** can be molecularly dissolved in these solvents, its emission can be seriously quenched by the free intramolecular motions through nonradiative decay. In comparison, with the increase of water content in water/DMSO and water/DMF mixtures, sharp enhancement and blueshift of emission were observed due to the intermolecular aggregate formation, which would revive the radiative channel in aqueous solution.^[^ ^9^ ^]^

Considering the large aromatic domain and the symmetrical molecular structure, we were curious to know whether the triphenylamine‐based cationic macrocycle **1** possessed the multiphoton absorption ability.^[^ ^10^ ^]^ The triphenylamine macrocycle **1∙4Cl** ^−^ could form supramolecular aggregate via its *π*‐conjugated skeleton, leading to the enhanced absorption properties. As expected, the fluorescence emission spectrum obtained upon excitation at 880 nm resembles the one upon excitation at 440 nm in water, indicative of the good two‐photon absorption property of **1∙4Cl** ^−^ (Figure S13, Supporting Information). In addition, no obvious absorbance change was found in the UV–vis spectra of **1** after light irradiation for more than 25 min (Figure S14, Supporting Information). Undoubtedly, these distinctive spectroscopic characteristics can simultaneously ensure the strong photoluminescence and good photostability under the biological environment.

**Figure 2:**
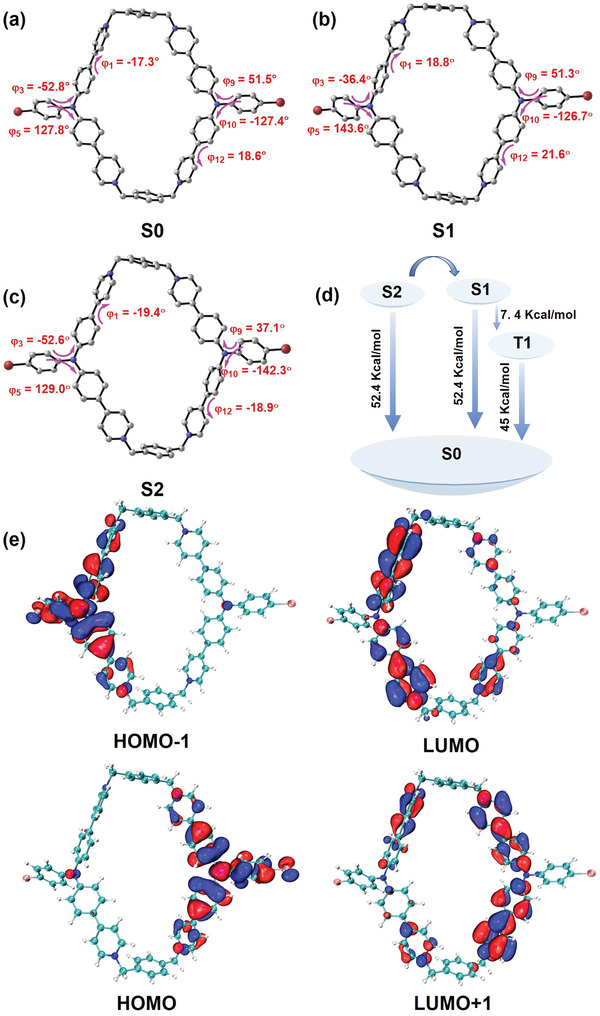
Optimized geometries and dihedral angles of **1∙4Cl** ^−^ in a) the ground state (S_0_), b) the first excited singlet state (S_1_), and c) the second excited singlet state (S_2_). Note that hydrogen atoms have been omitted for clarity. d) Energy‐level diagram of ground and excited states of **1∙4Cl** ^−^ e) Selected molecular orbitals of **1∙4Cl** ^−^ with the optimized ground‐state geometry in water.

To gain more insight into the photophysical properties of **1∙4Cl** ^−^, the corresponding molecular structures in the ground (S_0_) and excited (S_1_ and S_2_) states were optimized in by density functional theory (DFT) and time‐dependent density functional theory (TDDFT) calculations in water (**Figure** **2**a–c).^[^ ^11^ ^]^ Since the oscillator strength of S_2_ (1.938) is much larger than that of S_1_ (0.006), the simulated UV–vis absorption at 471.7 nm is predominately originated from the S_0_ → S_2_ excitation (Figure 2d and Figures S15 and S16 and Table S1, Supporting Information). Meanwhile, the proportions of Highest Occupied Molecular Orbital (HOMO) − 1 → Lowest Unoccupied Molecular Orbital (LUMO) and HOMO → LUMO + 1 excitation are also calculated as 45.8% and 47.8%, respectively.^[^ ^12^ ^]^ As for the HOMO − 1 → LUMO and HOMO → LUMO + 1 excitation, the electrons are initially localized at the *n* orbitals of nitrogen and bromide atoms as well as the *π* orbital of the phenyl group, and then transferred to the π* orbitals of the phenyl and pyridyl groups on the same side. Therefore, the low‐energy absorption band in the region of 350–750 nm can be assigned as a joint contribution of *n* → π* and *π* → π* transition (Figure 2e). Moreover, after screening the optimized geometries, it is found that compared to the S_0_ state, the selected dihedral angles of **1∙4Cl** ^−^ are basically unchanged in the S_1_ and S_2_ states, which can be attributed to the cyclization effect on the maintenance of structural stability and rigidity (Figure 2a–c and Figure S17 and Table S2, Supporting Information).

**Figure 3:**
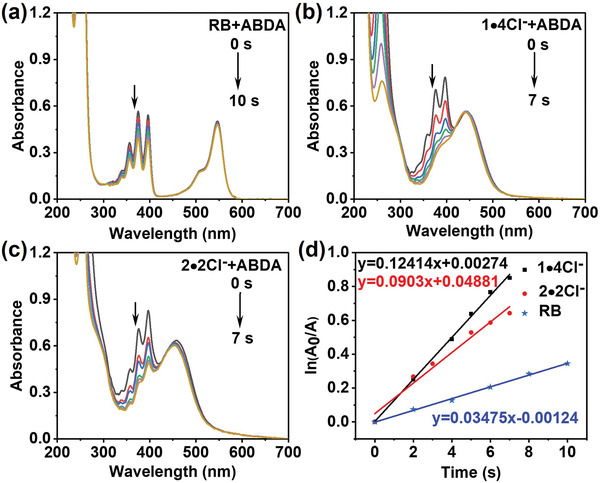
UV–vis absorbance intensity of ABDA in the presence of a) RB, b) **1∙4Cl** ^−^, c) **2∙2Cl** ^−^, and d) normalized degradation percentages of ABDA at 378 nm under white light irradiation (*λ* > 420 nm, 220 mW cm^−2^, [RB] = [**1∙4Cl** ^−^] = 1 × 10^−5^ m, [**2∙2Cl** ^−^] = 2 × 10^−5^ m, and [ABDA] = 5 × 10^−5^ m, H_2_O, 25 °C).

As a type of strong electron donors, triphenylamine is usually employed to elongate the *π*‐conjugation for modulating many photocatalysis processes.^[^ ^13^ ^]^ Therefore, besides the light absorption and photoluminescence properties, the photosensitized ^1^O_2_ generation ability of **1** was also investigated in different solvents by using 9,10‐anthracenediylbis(methylene)dimalonic acid (ABDA) as the detecting probe. As shown in Figure S18 in the Supporting Information, the UV–vis absorbance of ABDA obviously decreased in the presence of **1**, corresponding to the degradation of ABDA by ^1^O_2_ under light irradiation. Among all the examined solvents, the photodegradation of ABDA by **1** shows the fastest rate in water and most of ABDA can be decomposed upon light irradiation in only 90 s. Moreover, to quantitatively evaluate the ^1^O_2_ production efficacy, the ^1^O_2_ quantum yields of **1∙4Cl** ^−^ were tested using Rose Bengal (RB) as the standard.^[^ ^14^ ^]^ Compared to RB itself with 75% yield, the calculation results show that the ^1^O_2_ quantum yield of **1∙4Cl** ^−^ reaches nearly 130% in water (**Figure** **3**a,b and Figure S19, Supporting Information). For comparison, the degradation rate of ABDA became rather slower in the presence of the acyclic reference **2∙2Cl** ^−^ and accordingly, the ^1^O_2_ quantum yield of **2∙2Cl** ^−^ is determined as only 67% (Figure 3c,d and Figure S19, Supporting Information). These results substantiate that the macrocycle **1∙4Cl** ^−^ possesses good photodynamic effect and can be utilized as a photosensitizer accessible in aqueous solution.

**Figure 4:**
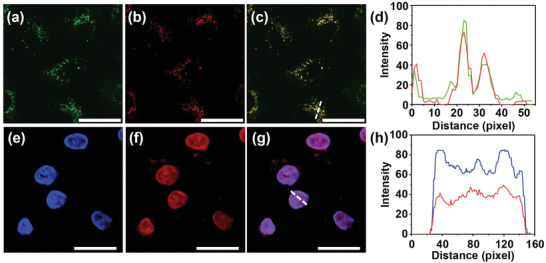
Lysosome and nucleus colocalization images in the living A549 cells upon coincubation with a) Lysotracker Green, b) **1∙4Cl** ^−^, c) merged image of (a) and (b), d) Z‐stacks image of (c) obtained from Zeiss Efficient Navigation (ZEN) package, e) DAPI, f) **1∙4Cl** ^−^, g) merged image of (e) and (f), and h) Z‐stacks image of (g) obtained from ZEN Lite package. The scale bar is 20 µm ([**1∙4Cl** ^−^] = 2.5 × 10^−6^ m, *λ* > 420 nm, 220 mW cm^−2^, 8 min).

On account of the excellent photophysical performance and ^1^O_2_ generation ability of **1∙4Cl** ^−^ in inanimate milieu, cell experiments were further performed to investigate its bioimaging and photodynamic abilities under physiological condition. First, the cytotoxicity toward the human carcinoma A549 cells was evaluated at different concentrations of **1∙4Cl** ^−^ by the cell‐counting kit‐8 assay. The results demonstrate that **1∙4Cl** ^−^ exhibited less dark toxicity within 10 × 10^−6^ m, while serious cell apoptosis was induced under light irradiation conditions in the presence of **1∙4Cl** ^−^ at only 1 × 10^−6^ m (Figure S20, Supporting Information). Taking the concentration at 5 × 10^−6^ m for example, more than 80% cells kept alive in the dark, but the relative cellular viability sharply declined to only 27% in the light treatment for 5 min. Next, the cell‐imaging ability of **1∙4Cl** ^−^ was examined using different commercially available organelle‐targeted agents. When **1∙4Cl** ^−^ was cocultured with A549 cells at the concentration of 2.5 × 10^−6^ m for 12 h, that bright red fluorescence could be readily observed in the cytoplasm and the confocal microscopic images show that **1∙4Cl** ^−^ is mainly distributed at lysosomes rather than mitochondria (**Figure** **4**a–d and Figure S21, Supporting Information). The Pearson correlation coefficients are accordingly calculated as 0.74 and 0.13 in lysosomes and mitochondria, respectively. Taken together, these results confirm that without light treatment, **1∙4Cl** ^−^ has preferential accumulate in the lysosomes of living cells.

**Figure 5:**
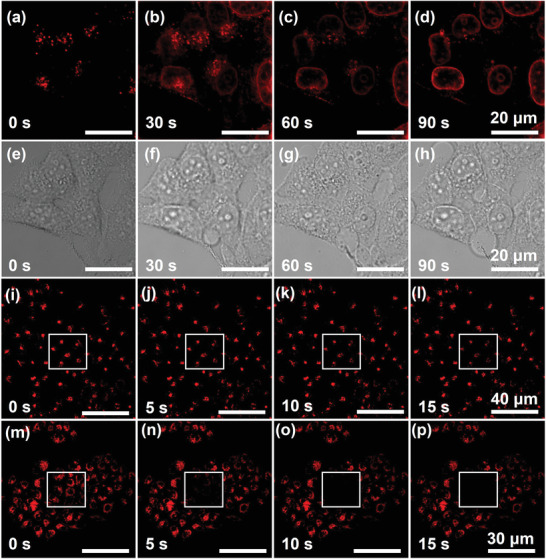
a–d) Confocal microscopic images of A549 cells upon incubation with **1∙4Cl** ^−^ for in situ monitoring of lysosome‐to‐nucleus translocation under light irradiation at different time intervals. e–h) The bright‐field images corresponding to the ones shown in (a)–(d). Confocal microscopic images of A549 cells upon incubation with i–l) **1∙4Cl** ^−^ and m–p) **2∙2Cl** ^−^ under continuous illumination for 15 s in the selected areas (white square).

After validating the lysosome‐targeting ability, the cell‐imaging properties of **1∙4Cl** ^−^ were comparatively studied with and without light irradiation. In dark, **1∙4Cl** ^−^ was located around the nucleus and could specifically stain lysosome in the living cells (Figure 4a–d). In contrast, after light irradiation at more than 420 nm for 8 min, strong red fluorescence exclusively appeared in the nucleus and perfectly overlapped with the blue region of nucleus‐targeted agent with a high Pearson's coefficient as 0.95 (4',6‐diamidino‐2‐phenylindole (DAPI), Figure 4e–h and Figure S22, Supporting Information). The time‐dependent in situ tracking experiments were also carried out to better observe the migration process from lysosomes to nucleus. In this case, the cell images were collected every 30 s in the same position. As depicted in **Figure** **5**a–d, red fluorescent dots initially gathered in lysosomes and both lysosomes and nuclei could be costained by extending the illumination time. The red fluorescence of **1∙4Cl** ^−^ finally located in the nuclei, accompanied by the ^1^O_2_‐induced cell apoptosis under light irradiation. Indeed, the ^1^O_2_ generation in the cellular environment could be further confirmed by the numerous bubbles shown on the cell surface (Figure 5e–h). Besides, the same migration process could also be observed in Hela cells. These results demonstrate the targeted translocation of **1∙4Cl** ^−^ among different organelles is common and reproducible in cancer cells (Figures S23 and S24, Supporting Information).

In the control experiments, although **2∙2Cl** ^−^ gave a quite similar cytotoxicity to **1∙4Cl** ^−^ at the relatively higher concentrations, much lower cell viability of the selected cell line was found upon coincubation of 2 equivalents of **2∙2Cl** ^−^ in dark (Figure S25, Supporting Information). Moreover, cell‐imaging experiments reveal that the red fluorescence of **2∙2Cl** ^−^ is distributed in the whole cytoplasm, and no migration phenomenon from lysosome to nucleus was observed for **2∙2Cl** ^−^ in the cell apoptosis process (Figure S26, Supporting Information). In addition, the photostability was also comparatively explored. The red fluorescence of **1∙4Cl** ^−^ could be detected after the laser irradiation for 15 s, whereas no fluorescence could be detected for **2∙2Cl** ^−^ under the same experimental condition (Figure 5i–p). These obtained results further illustrate the importance of cyclization effect, which is beneficial for enhancing the photostability in the bioimaging.

To shed light on the photocontrolled organelle‐specific translocation, the molecular binding behaviors were explored between **1∙4Cl** ^−^ and some bioactive substrates containing different numbers of negative charges, including singly and multiply charged nucleotides and G‐quadruplex (G4). These selected substrates are all the frequently encountered organic components and biomacromolecules enriched in the cell environment.^[^ ^15^ ^]^ As for the nucleoside monophosphate, no obvious spectral change was observed in UV–vis spectra, indicating that the electrostatic attraction with single charge is not sufficient to interact with **1** (Figure S27, Supporting Information). In addition, as the numbers of negative charges increased, the interaction of **1∙4Cl** ^−^ with adenosine triphosphate gave more visible changes than the one with adenosine diphosphate in the UV–vis absorbance (Figure S28, Supporting Information).

The enhanced association of **1∙4Cl** ^−^ with negatively charged nucleotides motivates us to explore its molecular interaction with nucleic acids. As we know, G4 is a secondary nucleic acid structure possessing guanine‐rich DNA sequences with high density of negative charges and has shown potent abilities in tuning diverse biological processes, especially in the tumor‐related gene regulation.^[^ ^16^ ^]^ In our case, Hum24, a 24‐membered oligonucleotide with the repeated subunit of human telomere, is chosen as a representative type of G4 family. As discerned from Figure S29a,b in the Supporting Information, obvious bathochromic shift was observed upon binding of Hum24 with **1∙4Cl** ^−^ in the UV–vis absorption and fluorescence emission spectra. In addition, the characteristic peaks in circular dichroism spectra also indicate the formation of antiparallel G‐quadruplex in the presence of K^+^.^[^ ^17^ ^]^ Comparatively, the negative peak at 260 nm underwent a large hypochromatic shift of 10 nm in the presence of **1∙4Cl** ^−^, also suggesting the strong interaction between **1∙4Cl** ^−^ and Hum24 (Figure S30, Supporting Information). Furthermore, spectroscopic titration experiments were performed to quantitively investigate the molecular recognition process. The binding stoichiometry of the association between **1∙4Cl** ^−^ and Hum24 is independently determined as 3:2 by UV–vis and fluorescence spectroscopy (Figure S31, Supporting Information), The binding constant could reach up to 10^6^ m ^−1^ order of magnitude by UV–vis spectroscopy (Figure S32, Supporting Information).^[^ ^18^ ^]^ It is known that a large number of G4 exist at the telomeres of cancer cells, owing to their infinite division activity in an uncontrolled way.^[^ ^19^ ^]^ In view of the strong binding of **1∙4Cl** ^−^ toward Hum24, it can be inferred that **1∙4Cl** ^−^ may have greater toxicity to cancer cells. To verify this assumption, human embryonic kidney 293T cells were employed to check the cytotoxicity effect of **1∙4Cl** ^−^ on normal cells. Indeed, the dark toxicity and phototoxicity of **1∙4Cl** ^−^ are much lower toward 293T cells than A549 cells (Figures S20 and S33, Supporting Information). Similarly, when the substrate screening was extended from G4 to common DNA, it is also found that the UV–vis absorbance in the range of 380–440 nm obviously decreased when calf thymus DNA (ctDNA) was used as model and added into the solution of **1∙4Cl** ^−^, further confirming its binding affinity with nucleic acids. The binding constant was accordingly measured in 10^7^ m ^−1^ order of magnitude by Scatchard plots (Figure S34, Supporting Information).^[^ ^20^ ^]^ Overall, these results demonstrate that although the molecular size of cationic macrocycle **1** is larger than other small molecular probes, four positive charges are intensively located on the molecular skeleton and **1** is prone to bind specific biomacromolecules with ordered structures and condensed negative charges mainly through electrostatic interaction, which may be contributed to its accumulation in the nucleus.

## 3 Conclusion

In conclusion, the triphenylamine‐based tetracationic macrocycle **1** is conveniently synthesized and its crystal structure is stabilized by the multiple intermolecular *π*‐stacking interactions. Benefitting from the cyclization effect, the obtained macrocycle **1** possesses more distinctive photophysical performance than the acyclic reference, including better photostability and photosensitizing ability, higher efficacy in ^1^O_2_ production, two‐photon absorption property, as well as stronger photoluminescence both in aqueous solution and solid state. Significantly, as revealed by the cell‐staining experiments, the macrocycle **1** can readily escape from lysosome to nucleus in the ^1^O_2_‐induced cell apoptosis process under light irradiation. It is also found that the *π*‐aromatic skeleton with high‐density positive charges in **1** can make very strong electrostatic attraction with G4 and other DNA, which can facilitate its preferential accumulation in the nuclei. Considering that the targeted translocation among different organelles is viewed as one of the main pathways for drug silencing and distribution, we can envision that our conformationally confined macrocycle with photocontrolled organelle‐specific translocation may pave a new avenue in creation of advanced optically active molecular probes and supramolecular nanomedicines.

## 4 Experimental Section

Detailed experimental procedures can be found in the Supporting Information.

## Conflict of Interest

The authors declare no conflict of interest.

## Supporting information

Supporting Information

Supporting Information

Supplemental Movie 1

## Data Availability

The data that support the findings of this study are available in the supplementary material of this article.
