# Supplementary material for: Conformationally Confined Emissive Cationic Macrocycle with Photocontrolled Organelle‐Specific Translocation
Source: Adv Sci (Weinh). 2022 Jun 17;9(23):2201962. doi: 10.1002/advs.202201962 (PMC9376817; doi:10.1002/advs.202201962)
Supplement: Supplementary file 1 — Supporting Information [file ADVS-9-2201962-s001.pdf]

ADVANCED SCIENCE
Open Access

Supporting Information

for *Adv. Sci.*, DOI 10.1002/advs.202201962

Conformationally Confined Emissive Cationic Macrocycle with Photocontrolled Organelle-Specific Translocation

Xiaoyun Dong, Xianyin Dai, Guorong Li, Ying-Ming Zhang, Xiufang Xu and Yu Liu**

Supporting Information

Conformationally Confined Emissive Cationic Macrocycle with Photo-Controlled Organelle-Specific Translocation

Table of Contents

**Charts S1–S2.** Synthetic routes	Page 6
**Figures S1–S5.** Structural Characterization of **1** and **2**	Page 7
**Figure S6.** Crystal structures of **1**	Page 10
**Figure S7.** Uv-vis spectra of **1**	Page 10
**Figures S8–S9.** Fluorescence emission spectra of **1**	Page 11
**Figures S10–S11.** Quantum yields of **1**	Page 11
**Figure S12.** Fluorescence emission of **1** in different solvents	Page 12
**Figure S13.** Two-photo fluorescence emission spectra of **1**	Page 13
**Figure S14.** Uv-vis absorbance of **1** under light irradiation	Page 13
**Figure S15.** Simulated UV-vis spectrum of **1**	Page 14
**Figure S16.** Contributions of individual excitations	Page 14
**Table S1**. Computed eight excited states of **1**	Page 14
**Figure S17.** The dihedral angles of **1**	Page 16
**Table S2**. Computed twelve dihedral angles of **1**	Page 16
**Figure S18**. Singlet oxygen generation of **1**	Page 17
**Figure S19**. $^1O_2$ quantum yield calculation of **1**	Page 17
**Figure S20**. Cell viability of A549 cells with **1**	Page 18
**Figure S21**. Mitochondria colocalization images	Page 18
**Figure S22**. Pearson's correlation coefficient	Page 18
**Figure S23**. Colocalization images	Page 19
**Figure S24**. Pearson's correlation coefficient	Page 19
**Figure S25**. Cell viability of A549 cells with **2**	Page 20
**Figure S26**. Confocal laser scanning microscopic images of **2**	Page 20
**Figure S27**. Uv-vis absorbance spectra of **1** with nucleosides	Page 21

Figure S28. Uv-vis absorbance spectra of **1** with ATP Page 21
Figure S29. Uv-vis and fluorescence spectra of **1·4Cl‾** and Hum24 Page 22
Figure S30. CD spectra of Hum24 Page 22
Figure S31. Job plots of **1** with Hum24 Page 22
Figure S32. Uv-vis titration spectra of **1·4Cl‾** Page 23
Figure S33. Cell viability of 293T cells with **1** Page 23
Figure S34. Uv-vis absorbance spectra of **1** with DNA Page 24

Chemical experimental details

All the reagents and materials were purchased from commercial suppliers were used without further purification unless otherwise stated. Thin-layer chromatography (TLC) and column chromatography were performed on silica gel. The UV-Vis absorption and fluorescence emission spectra were performed at 25 °C in acetonitrile and H_2O, and the Nuclear magnetic resonance (NMR) experiments were performed in CD_3CN. UV-Vis absorption spectra were recorded in a conventional rectangular quartz cell (1 cm × 1 cm) on a spectrophotometer. Fluorescence spectra were measured in a rectangular quartz cell (1 cm × 1 cm) on a spectrometer. NMR spectra were recorded on Bruker Avance III 400 MHz spectrometers with working frequencies of 400 MHz for ^{1}H NMR and 100 MHz for ^{13}C NMR, respectively. Chemical shifts are reported in ppm relative to the signals corresponding to the residual non-deuterated solvents (CD_3CN: δ_H = 1.96 ppm and δ_C = 1.3 and 118.3 ppm). CCK8 assay was detected by Microplate Reader (American BioTek Synergy 4) and Microsoft 2019 and OriginPro 2020 were used for data analysis.

Crystallographic characterization

All crystallographic data are available from the Cambridge Crystallographic Data Center (CCDC).

The compound **1·4PF$_6$‾** was dissolved in acetonitrile and the solution was filtrated through a 0.45 μm filter into a small glass tube. Then, a small amount of water was added to the above solution until the trace amounts of precipitation produced. The mixed solution was evaporated naturally under the room temperature for about 4 days

to obtain the organic-yellow crystals. The data was collected at 113.15 K on Rigaku 007 Saturn 70 equipped with Confocal Max-Flux optical system, and the CCDC number is 2074826.

Singlet oxygen ($^1O_2$) determination under white light irradiation

The $^1O_2$ generation capacity of **1·4Cl¯** was determined with the 9,10-anthracenediylbis(methylene)dimalonic acid (ABDA) as the probe and Rose Bengal (RB) as standard using Uv-vis spectroscopy. The Uv-vis absorbance intensity of ABDA gradually decreased with the generation of $^1O_2$. Singlet oxygen quantum yield was calculated by the following equation:

$$\emptyset_{\mathrm{G}} = \emptyset_{\mathrm{ST}} \frac{k_{\mathrm{G}}}{k_{\mathrm{ST}}} / \frac{A_{\mathrm{G}}}{A_{\mathrm{ST}}}$$

where k_{G} and k_{ST} represent the decomposition rate constants of ABDA with photosensitizer (G) and RB (ST), respectively. A_{G} and A_{ST} represent the light absorbed by photosensitizer (G) and ST, respectively, which are determined by integration of the areas under the absorption bands in the wavelength range of 400–600 nm. Φ_{ST} is the $^1O_2$ quantum yield of ST, which is 0.75 in water.

Cell experiments

Cell culture. Human carcinoma A549 cell line and human embryonic kidney 293T cell line were obtained from Institute of Basic Medical Science, Chinese Academy of Medical Science. A549 cells were cultured in a cell incubator with Ham's F12 nutrient medium containing 10% fetal bovine serum and 1% penicillin−streptomycin under 5% CO_2 at 37 °C. Additionally, 293T cells were cultured in a cell incubator with a DMEM high-glucose nutrient medium containing 10% fetal bovine serum and 1% penicillin−streptomycin in a humidified standard under 5% CO_2 at 37 °C.

Confocal fluorescence imaging. A549 cells were seeded onto a laser confocal Petri dish and cultivated for 24 h. Then, the **1·4Cl¯** were added into the dish and incubated with the cells for different durations of 12 h. Afterward, the culture medium was removed and washed with PBS (0.01 M) three times, then, the fresh culture medium was added. Subsequently, Mito-Tracker Green and Lysotracker Green was added to stain the mitochondria at 37 °C for 30 min, respectively. And the nucleus was stained

by DAPI for 30 min at 37 °C. After repeated washing with PBS three times, the cells were observed directly via CLSM. For Mito-Tracker Green and Lysotracker Green, the excitation filter was 488 nm, and the emission was 510−540 nm. For DAPI, the excitation filter was 405 nm, and the emission was 420−450 nm. For **1·4Cl¯**, the excitation filter was 405 nm, and the emission was 600−700 nm. The fluorescence emission spectrum of **1·4Cl¯** exhibited broad emission band ranging from 470 to 750 nm. In order to avoid overlapping with the fluorescence of Lysotracker Green and Mitotracker Green, only the fluorescence in the 600-700nm region was collected during confocal microscopic images.

Cell cytotoxicity. The A549 cells and 293T cells were seeded into a 96-well plate for 24 h and then the medium was replaced with 100 μL fresh culture medium containing **1·4Cl¯** at different concentrations. Then, the cells were incubated with **1·4Cl¯** in the dark for 24 h. After that, the white light irradiation (λ > 420 nm, 220 mW/cm^2) was applied for 2.5 min and 5 min, respectively. Then, the cells were continuously cultured for another 1 h. The culture medium was removed after repeated washing with PBS three times, then the fresh culture medium was added. The cell viability was evaluated by the cell-counting kit-8 (CCK-8) assay according to the kit instruction. The plate was then read by a microplate reader at a wavelength of 450 nm. Three values are taken for each concentration condition. All the data were presented as the mean ± standard deviation.

Statistical Analysis. Three replicates of each experiment were performed, and values herein stand for means ± standard deviations (SD). Differences between groups were compared by a one-way analysis of variance test (p < 0.05). All statistical tests were performed using the SPSS software package (ver. 20, IBM, USA).

Scatchard plots. To further investigate the interactions between **1·4Cl¯** and G-quadruplex, the Uv-vis titration spectra obtained in the text were used to establish Scatchard plots:

$$\frac{r}{C_f} = nK_a - K_a r$$

or $$r = \frac{nK_aC_f}{(1+K_aC_f)}$$

where r is the number of moles of bound **1·4Cl¯** per mole of G-quadruplex; n is the number of ligand-binding sites on the G-quadruplex; K_a is the binding constant; and C_f is the free **1·4Cl¯** in the **1·4Cl¯**/G-quadruplex mixture, respectively.[S1-S2]

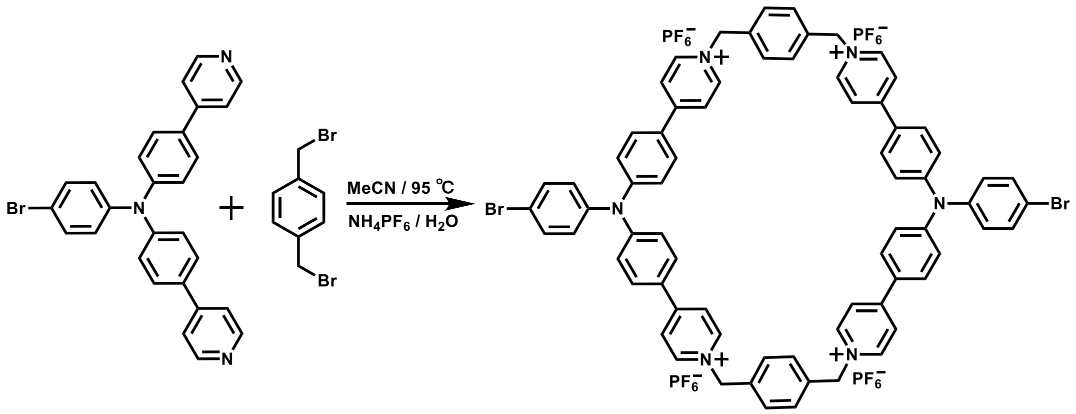

I **1·4PF$_6$¯**

Chart S1. Synthesis of 1·4PF$_6$¯

The compound **I** was synthesized according to the previous reports.[S3] The compound **I** (100 mg) was dissolved in acetonitrile (100 mL), and then 1,4-bis(bromomethyl)benzene (54 mg) was added the solution. The mixed solution was refluxed for 3 days under the argon at 95 °C. The reaction mixture was then cooled to room temperature and the precipitate was collected. The precipitate was washed using acetonitrile to obtain red solid. The collected solid was dissolved in H_2O and CH_3OH, and NH_4PF_6 was added in the solution. The solution was stirred until the precipitate was not formed. The precipitate was collected through the filtration and purified by column chromatography using acetonitrile and CH_2Cl_2 as eluent to obtain orange solid (yield: 8%). ^{1}H NMR (400 MHz, CD_3CN) δ 8.62–8.58 (m, 8H), 8.20–8.16 (m, 8H), 7.90–7.86 (m, 8H), 7.64 (d, J = 8.8 Hz, 4H), 7.61 (s, 8H), 7.27 (d, J = 8.9 Hz, 8H), 7.17 (d, J = 8.8 Hz, 4H), 5.70 (s, 8H).

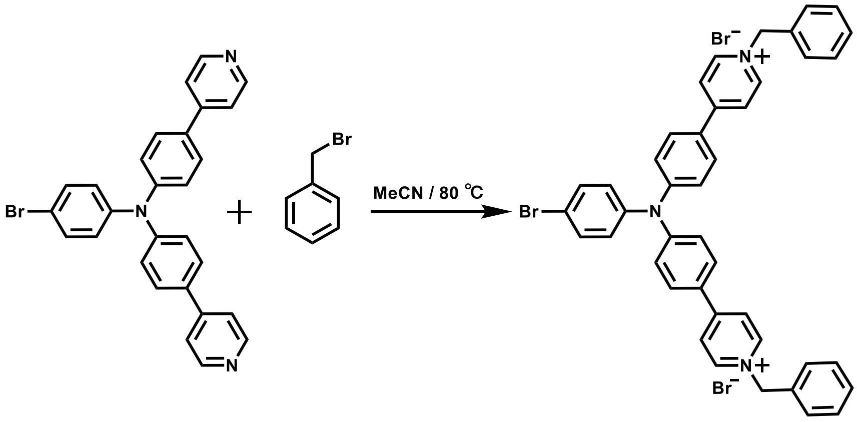

I **2·2Br⁻**

Chart S2. Synthesis of 2·2Br⁻

The compound **I** (100 mg) was dissolved in acetonitrile (50 mL), and then benzyl bromide (2 mL) was added to the solution. The mixed solution was refluxed for overnight under the argon at 80 °C. The reaction mixture was cooled to room temperature and the precipitate was collected. The precipitates were washed with a large amount of the acetonitrile to obtain **2·2Br⁻** as the orange solid (yield: 58%). The **2·2Cl⁻** was obtained through ion exchange process. ^{1}H NMR (400 MHz, D_2O) δ 8.82–8.72 (d, 4H), 8.24 (d, *J* = 6.2 Hz, 4H), 7.91 (d, *J* = 8.5 Hz, 4H), 7.61 (d, *J* = 8.0 Hz, 4H), 7.52 (m, 10H), 7.34 (d, *J* = 8.3 Hz, 4H), 7.18 (d, 4H), 5.76 (s, 4H).

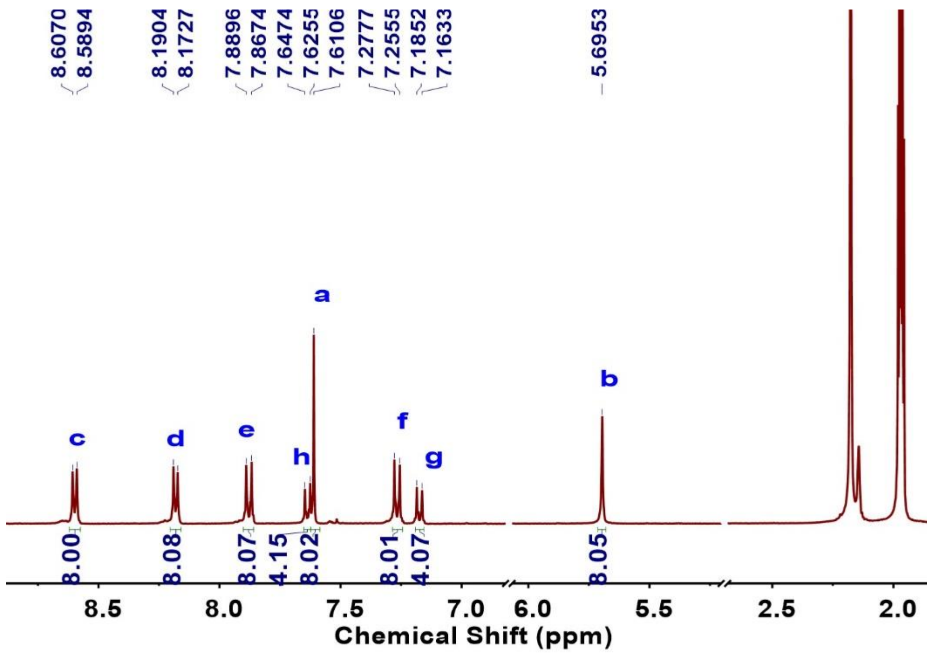

Figure S1. ^{1}H NMR spectrum of **1·4PF$_6$$^{-}$** (400 MHz, CD_3CN, 25 °C).

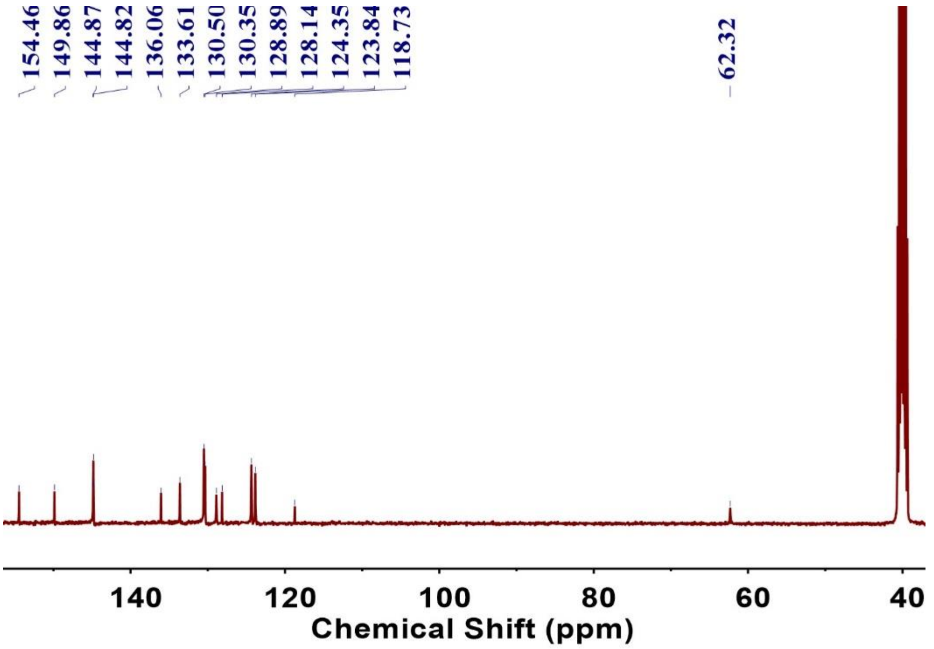

Figure S2. ^{13}C NMR spectrum of **1·4PF$_6$$^{-}$** (100 MHz, DMSO-$d_6$, 25 °C).

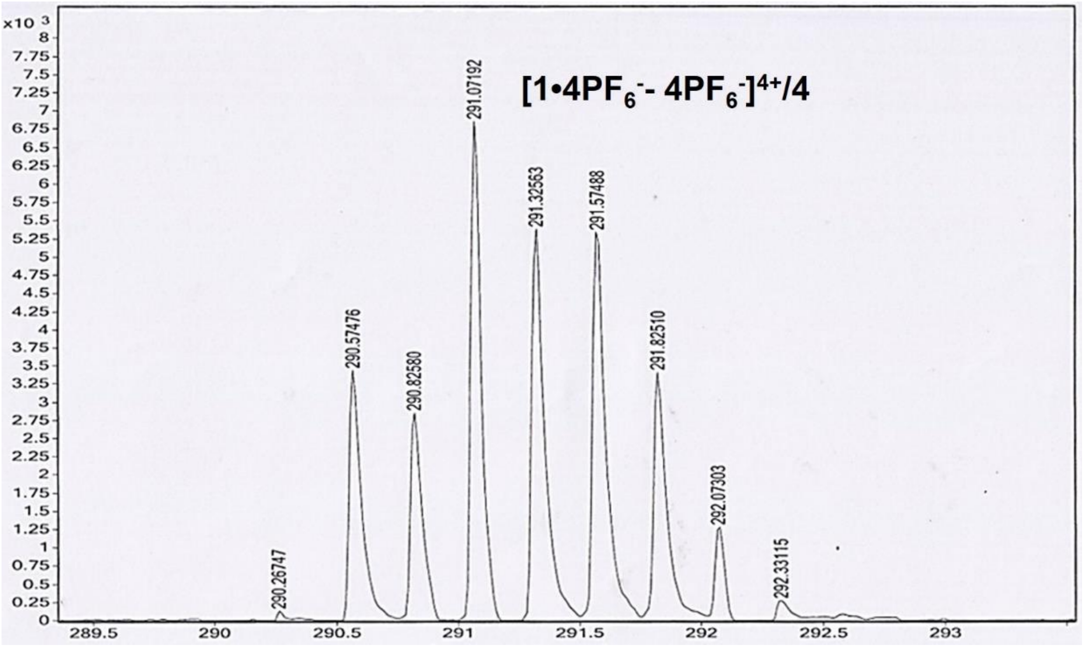

Figure S3. FT-MS spectrum of **1·4PF$_6$$^{-}$**.

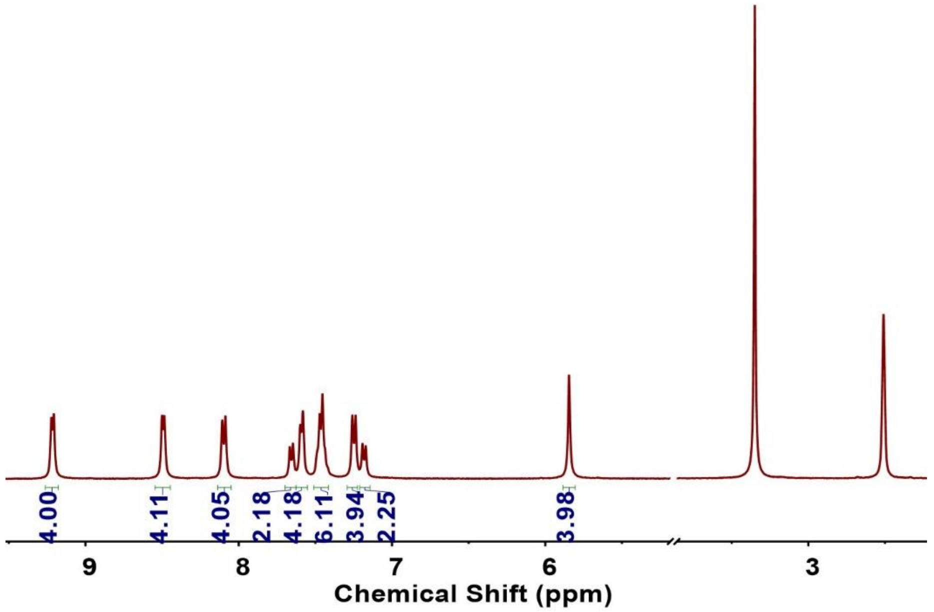

Figure S4. ^{1}H NMR spectrum of **2·2Br^{-}** (400 MHz, DMSO-d_6, 25 °C).

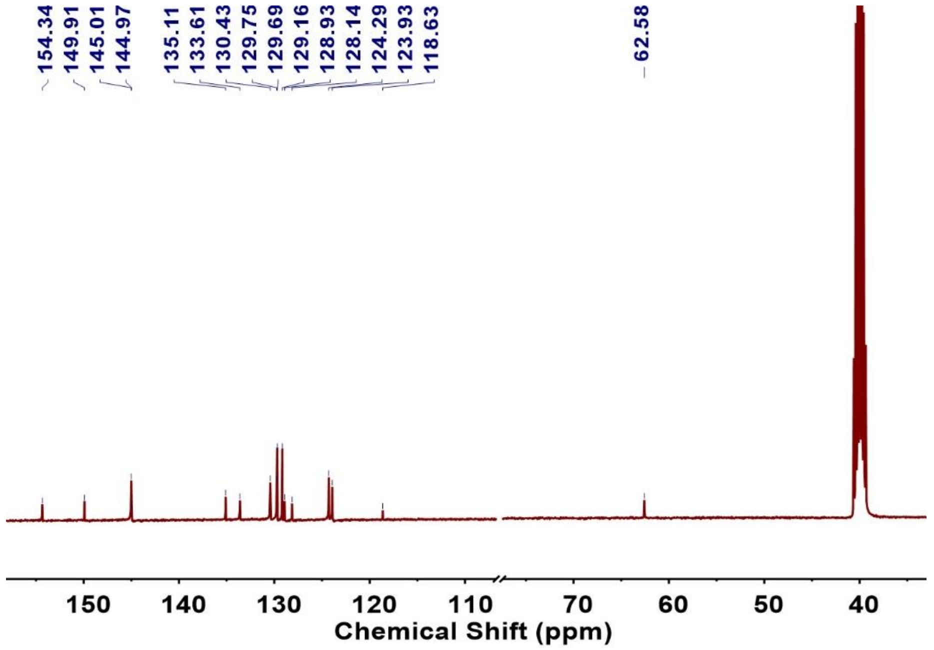

Figure S5. ^{13}C NMR spectrum of **2·2Br^{-}** (100 MHz, DMSO-d_6, 25 °C).

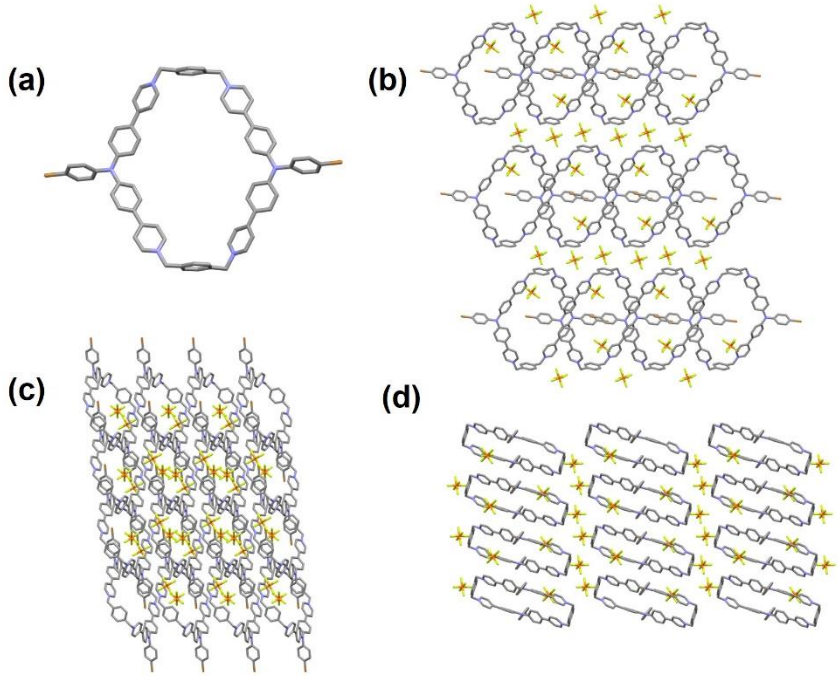

Figure S6. The solid structure of **1·4PF$_6$¯** obtained from X-ray crystallography. (a) single crystal structure unit; and the stacking mode along (b) a; (c) c; (d) b axles, respectively.

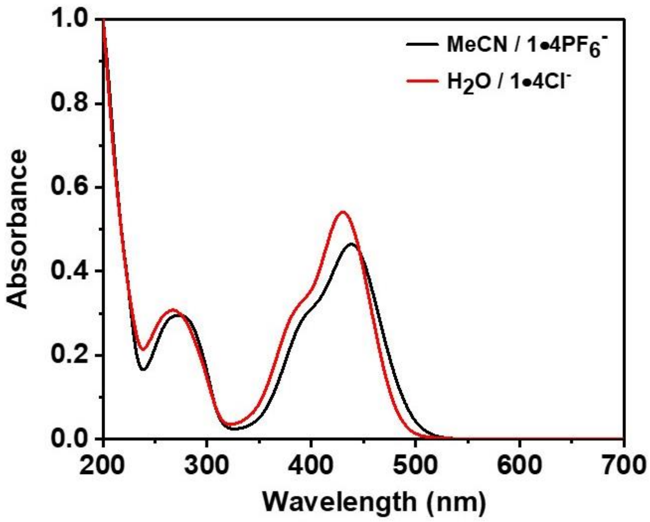

Figure S7. Uv-vis spectra of **1·4PF$_6$¯** in acetonitrile and **1·4Cl¯** in H_2O ([**1·4PF$_6$¯**] = [**1·4Cl¯**] = 1 × 10^{-5} M).

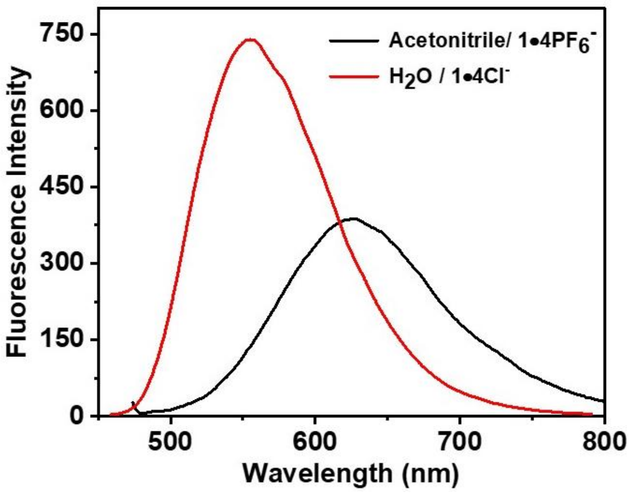

Figure S8. Fluorescence emission spectra of **$1\cdot4PF_6^-$** in acetonitrile and **$1\cdot4Cl^-$** in H_2O ([**$1\cdot4PF_6^-$**] = [**$1\cdot4Cl^-$**] = 1×10^{-5} M).

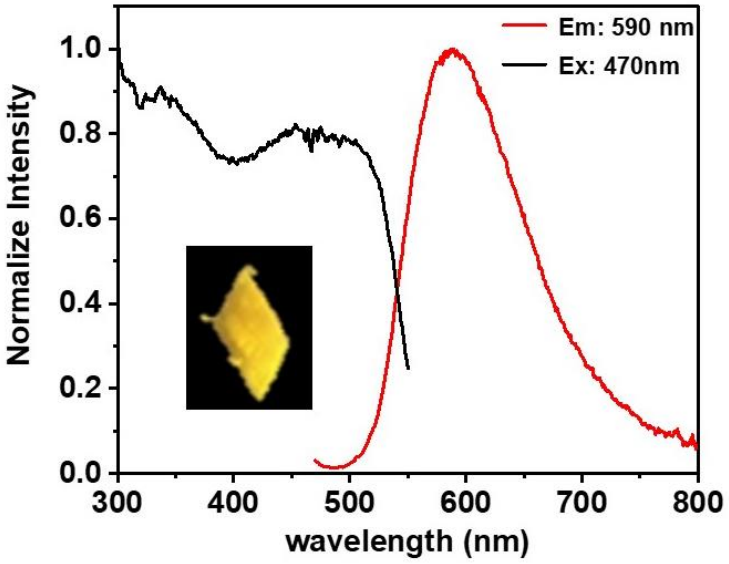

Figure S9. Fluorescence emission spectrum of **$1\cdot4PF_6^-$** in the solid state.

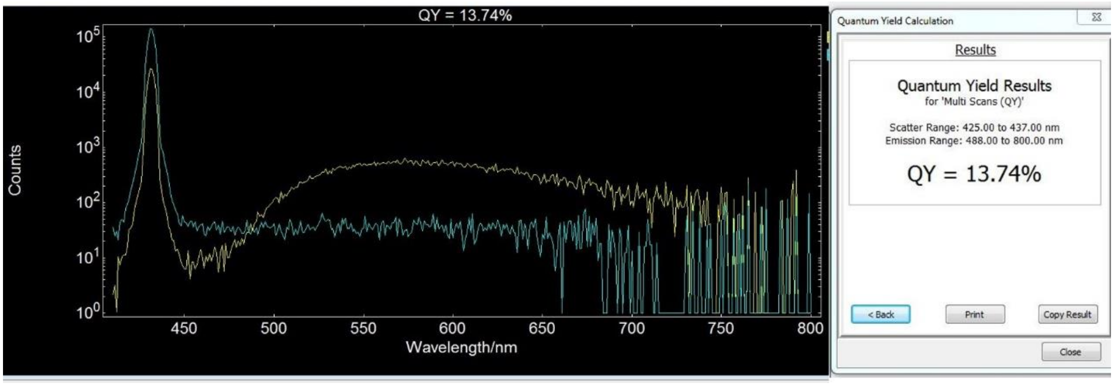

Figure S10. Quantum yield of **$1\cdot4Cl^-$** in water ([**$1\cdot4Cl^-$**] = 1×10^{-5} M).

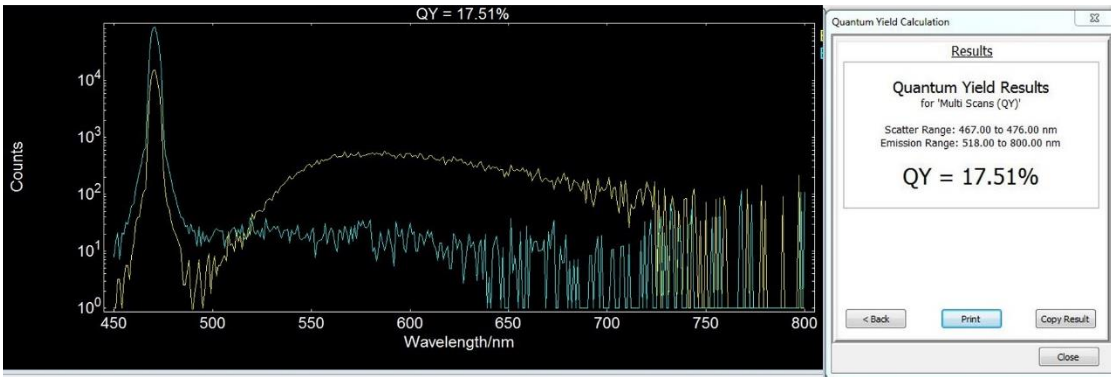

Figure S11. Quantum yield of **1·4PF$_6$¯** in the solid state.

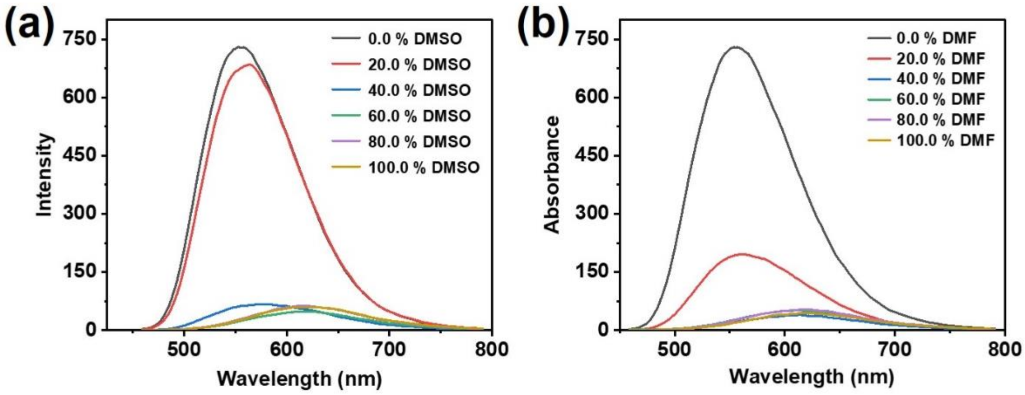

Figure S12. Fluorescence intensity of **1·4Cl¯** in different mixed solvents containing DMSO and DMF ([**1·4Cl¯**] = 1 × 10^{-5} M).

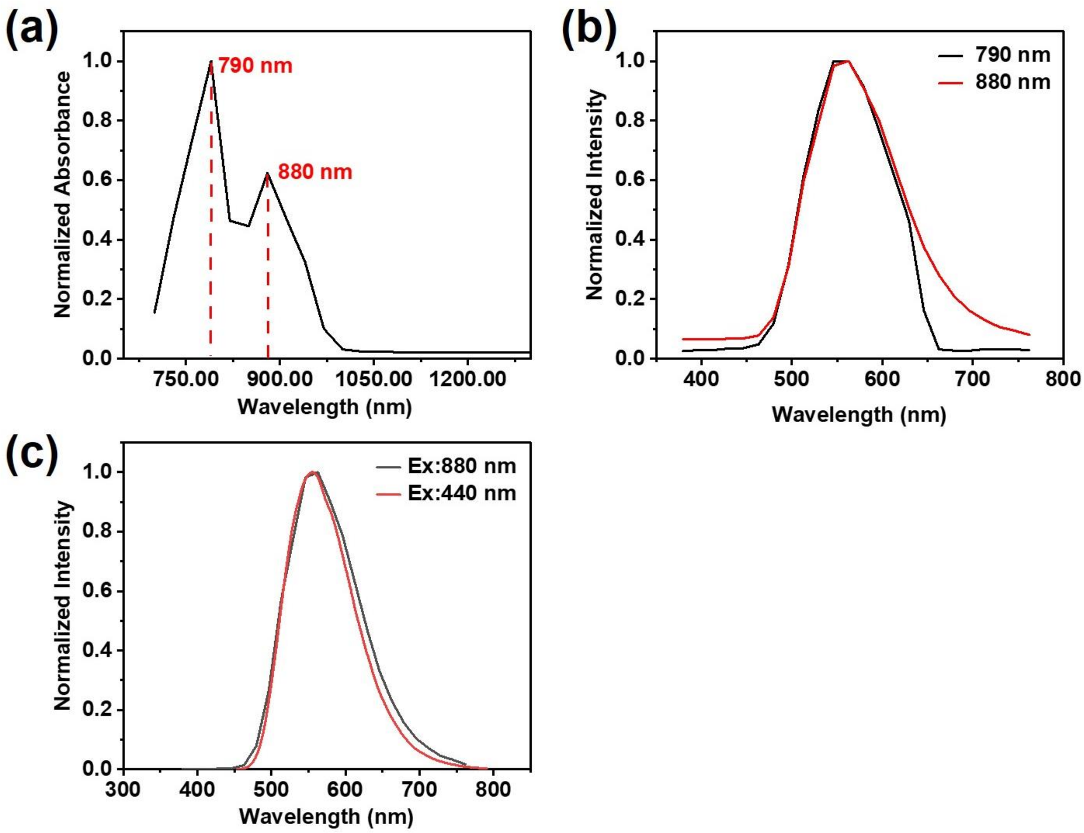

Figure S13. (a) UV-vis absorption spectrum, (b) Fluorescence emission spectra of **1·4Cl¯** upon excitation at 790 and 880 nm, and (c) Fluorescence emission spectra of **1·4Cl¯** upon excitation at 440 and 880 nm, respectively ([**1·4Cl¯**] = 1 × 10^{-5} M).

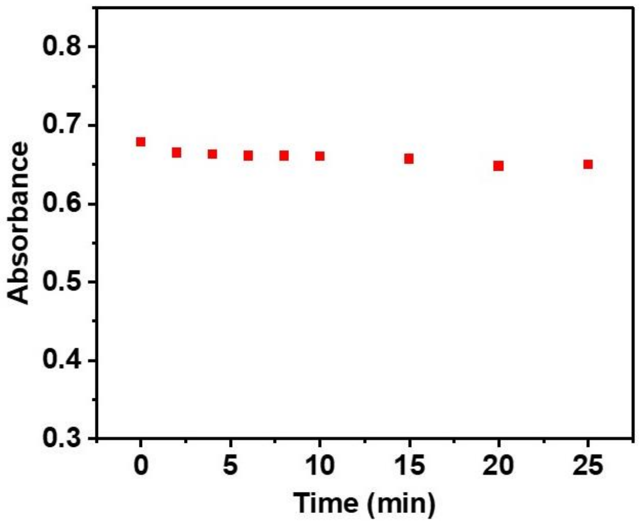

Figure S14. Uv-vis absorbance changes of **1·4Cl¯** versus different illumination time (λ > 420 nm, 220 mW/cm^2, [**1·4Cl¯**] = 1 × 10^{-5} M).

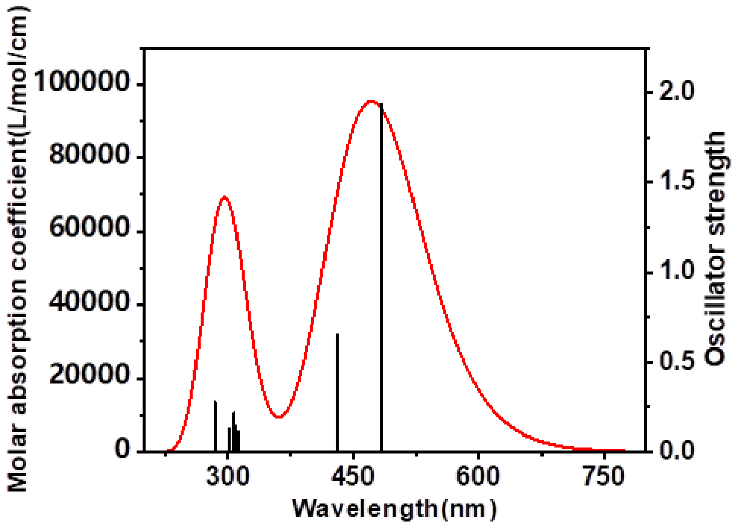

Figure S15. Simulated UV-vis spectrum of **1·4Cl**$^-$ computed by TDDFT/SMD using water as the solvent with D3(BJ) dispersion correction.

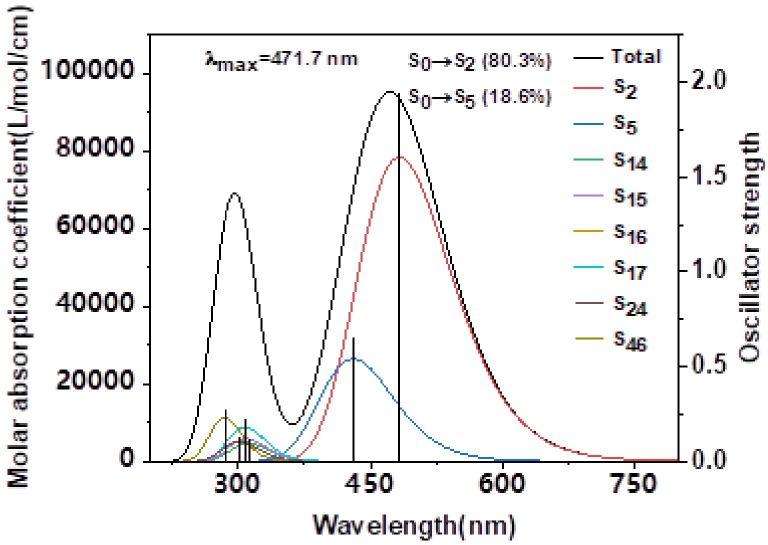

Figure S16. Contribution of individual excitations to the simulated Uv-vis spectrum of **1·4Cl**$^-$ with the values of oscillator strength larger than 0.1.

Table S1. The eight excited states (S_n, f > 0.1) among different HOMO (H) and LUMO (L) of **1·4Cl**$^-$ computed by TDDFT/SMD using water as the solvent.

S_n	Excitation (Coefficient > 0.2)	Vertical excitation wavelength (nm)	f
S_2	H-1→L (-0.48)	482.9	1.938

	H→L+1 (0.49)		
S_5	H-1→L+2 (0.46)	430.0	0.652
	H-1→L+3 (0.24)		
	H→L+2 (0.33)		
	H→L+3 (-0.34)		
S_{14}	H-3→L (-0.32)	312.1	0.113
	H-2→L+1 (0.38)		
	H→L+4 (0.21)		
S_{15}	H-4→L (-0.24)	309.3	0.143
	H-2→L+1 (0.21)		
	H→L+4 (-0.24)		
	H→L+9 (-0.22)		
	H→L+11 (0.24)		
S_{16}	H-3→L (0.24)	308.2	0.116
	H-1→L+4 (0.27)		
	H-1→L+9 (0.21)		
	H-1→L+10 (-0.23)		
	H-1→L+11 (0.28)		
S_{17}	H-4→L (0.46)	307.8	0.216
	H→L+4 (-0.21)		
	H→L+6 (0.27)		
S_{24}	H-6→L+1 (-0.30)	301.4	0.127
	H→L+10 (-0.21)		
	H→L+13 (0.36)		
S_{46}	H-5→L+2 (0.37)	284.7	0.278
	H-4→L+3 (-0.33)		
	H-1→L+8 (0.26)		

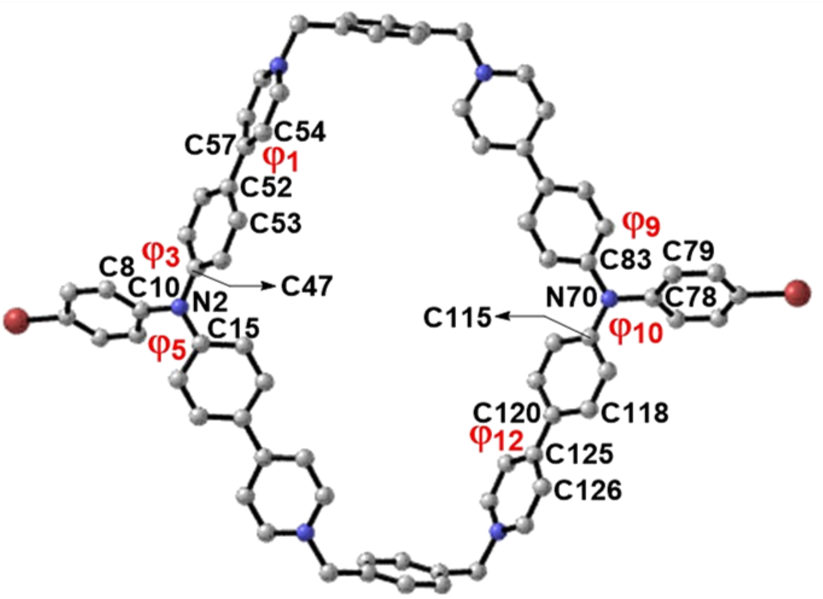

Figure S17. Reference numbers for the labeled atoms of macrocycle **1·4Cl¯**.

Table S2. Twelve dihedral angles of macrocycle **1** in the $S_{0\text{–}2}$ states.

	ϕ_1	ϕ_2	ϕ_3	ϕ_4	ϕ_5	ϕ_6
	C53-C52 -C57-C54	C10-N2 -C47-C55	C8-C10 -N2-C47	C10-N2 -C15-C16	C8-C10 -N2-C15	C18-C20 -C25-C26
S_0	-17.3°	148.3°	-52.8°	146.4°	127.8°	29.5°
S_2	-19.4°	146.9°	-52.6°	147.5°	129.0°	31.7°
S_1	18.8°	140.7°	-36.4°	145.7°	143.6°	24.3°
	ϕ_7	ϕ_8	ϕ_9	ϕ_{10}	ϕ_{11}	ϕ_{12}
	C89-C88 -C93-C100	C78-N70 -C83-C91	C79-C78 -N70-C83	C79-C78 -N70-C115	C78-N70 -C115-C116	C118-C120 -C125-C126
S_0	-30.3°	31.3°	51.5°	-127.4°	32.3°	18.6°
S_2	-24.3°	35.6°	37.1°	-142.3°	40.0°	-18.9°
S_1	-29.0°	32.3°	51.3°	-126.7°	31.4°	21.6°

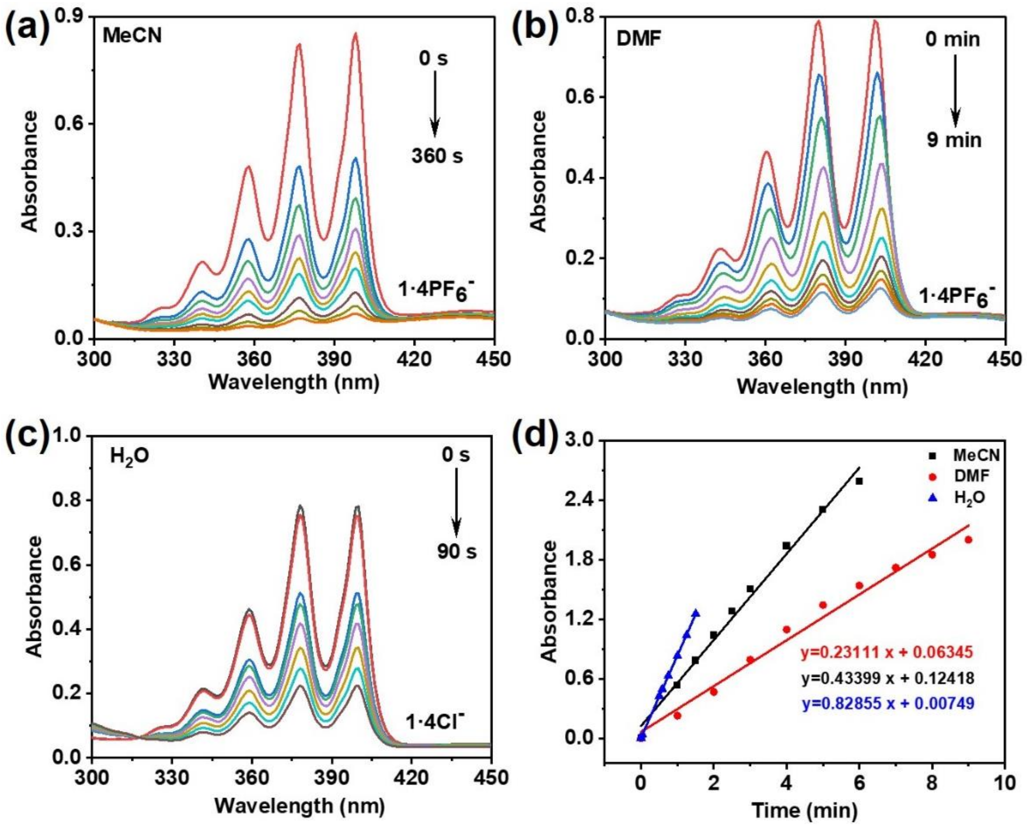

Figure S18. Singlet oxygen generation of **1** in different solvents. UV-vis spectra of ABDA in the presence of **1·4PF$_6$¯** under white-light irradiation in (a) acetonitrile and (b) DMF. (c) UV-vis spectra of ABDA in the presence of **1·4Cl¯** under white-light irradiation in water. (d) Normalized degradation percentages of ABDA at 378 nm in the presence of **1·4PF$_6$¯** or **1·4Cl¯** (λ > 420 nm, 220 mW/cm^2, [**1·4PF$_6$¯**] = [**1·4Cl¯**] = 1 × 10^{-6} M and [ABDA] = 6.67 × 10^{-5} M).

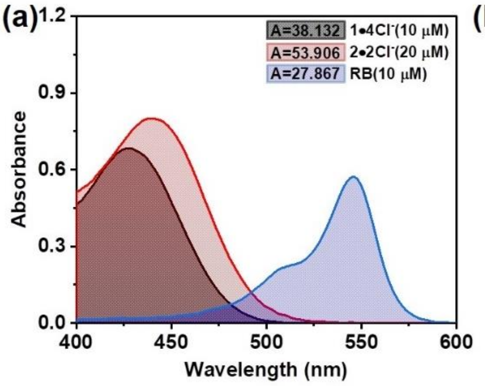

(b)

Cpd	$^1O_2$ quantum yield
RB (10 μM)	0.75
1•4Cl$^-$ (10 μM)	1.298
2•2Cl$^-$ (20 μM)	0.668

Figure S19. (a) Uv-vis spectra of RB, **1·4Cl¯**, and **2·2Cl¯** between 400 and 600 nm; (b) $^1O_2$ quantum yields of **1·4Cl¯**, **2·2Cl¯** with the RB as the standard ([**1·4Cl¯**] = 1 × 10^{-5} M, [**2·2Cl¯**] = 2 × 10^{-5} M, and [RB] = 1 × 10^{-5} M).

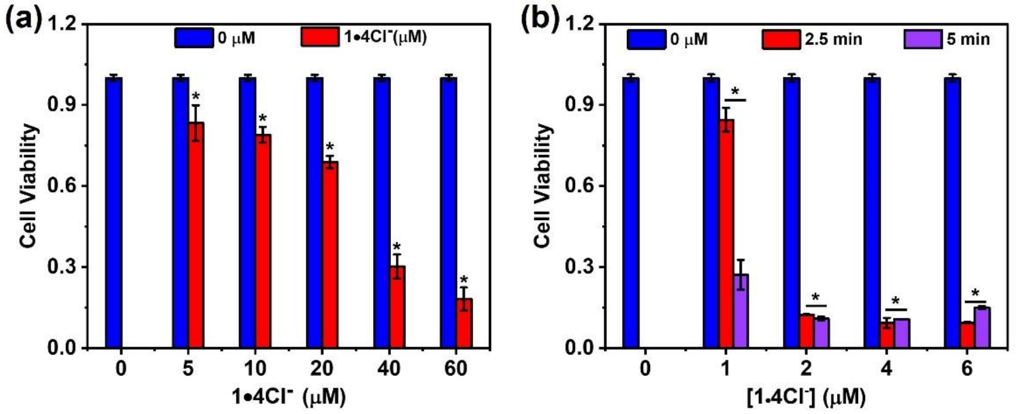

Figure S20. A549 Cell viability at different concentrations of **1·4Cl¯** (a) without and (b) with white-light irradiation (λ > 420 nm, 220 mW/cm^2). Three independent experiments, with the data indicating mean ± SD. Asterisk * indicates a significant difference between the control group (0 μM **1·4Cl¯** without irradiation and with irradiation) and treated group (**1·4Cl¯** without irradiation and with irradiation) ($p < 0.05$).

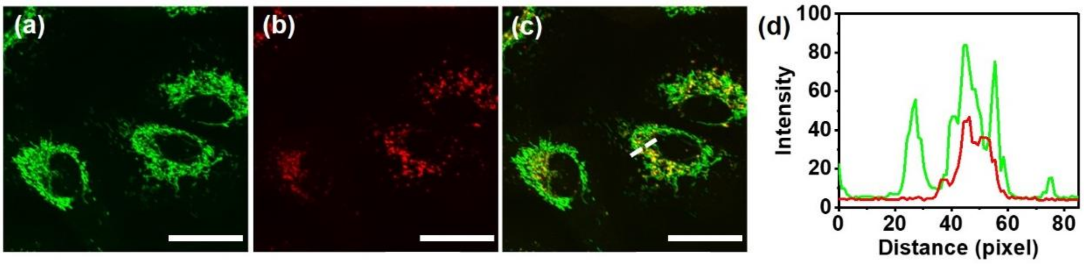

Figure S21. Mitochondria colocalization images in the living A549 cells upon co-incubation with (a) Mitotracker Green, (b) **1·4Cl¯**, (c) merged image, and (d) Z-stacks image obtained from ZEN Lite. The scale bar is 20 μm.

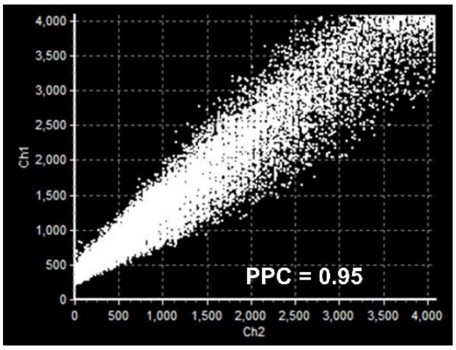

Figure S22. Pearson's correlation coefficient obtained from the scatter plot.

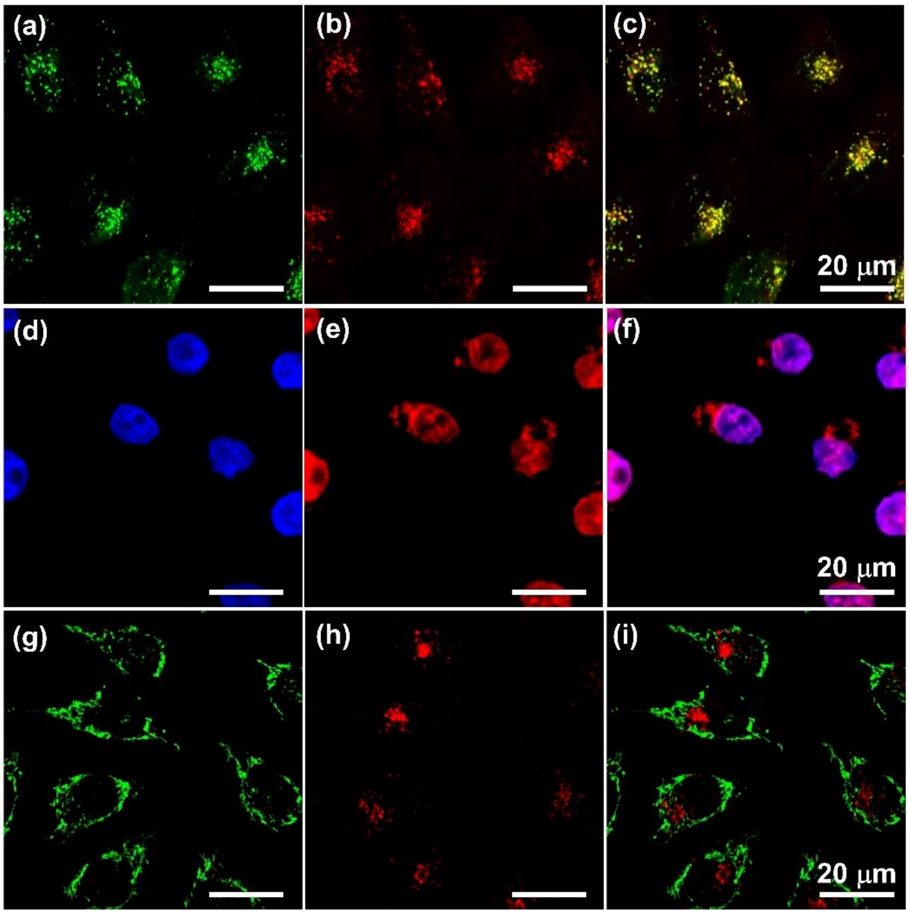

Figure S23. Lysosome, mitochondrion and nucleus colocalization images in the living (a-c, g-i) and dead (d-f) Hela cells upon co-incubation with (a) Lysotracker Green, (b) **1·4Cl¯**, (c) merged image of (a) and (b), (d) DAPI, (e) **1·4Cl¯**, (f) merged image of (d) and (e), and (g) Mitotracker Green, (h) **1·4Cl¯**, (i) merged image of (g) and (h). The scale bar is 20 μm ([**1·4Cl¯**] = 2.5 μM, λ > 420 nm, 220 mW/cm^2, 8 min).

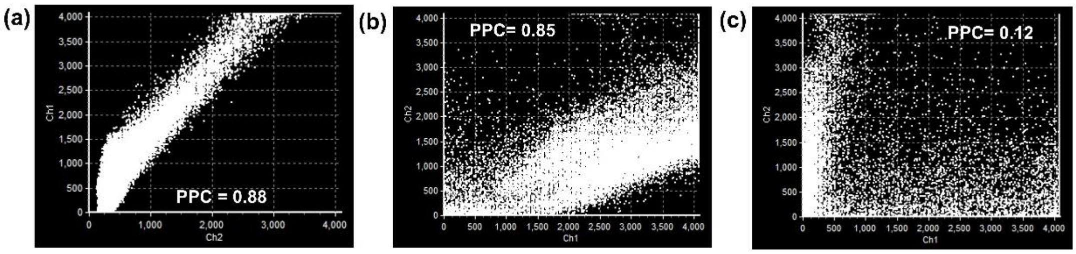

Figure S24. Pearson's correlation coefficients obtained from the scatter plot. (a) Lysosomes, (b) nucleus and (c) mitochondria.

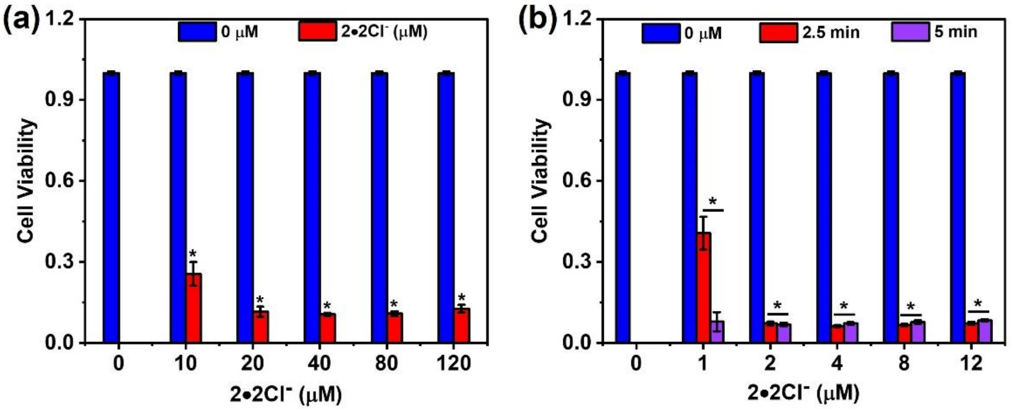

Figure S25. Cell viability of A549 cells at different concentrations of **2·2Cl¯** (a) without and (b) with light irradiation (λ > 420 nm, 220 mW/cm^2). Three independent experiments, with the data indicating mean ± SD. Asterisk * indicates a significant difference between the control group (0 μM **2·2Cl¯** without irradiation and with irradiation) and treated group (**2·2Cl¯** without irradiation and with irradiation) (p < 0.05).

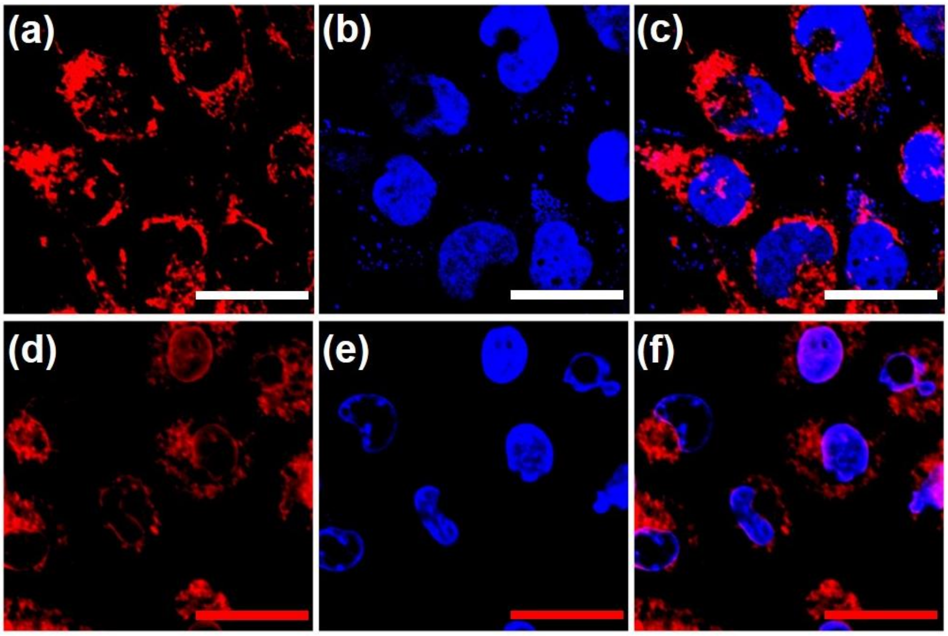

Figure S26. Confocal laser scanning microscopic images of **2·2Cl¯** in the A549 cells (a–c) without and (d–f) with white-light irradiation: (a, d) **2·2Cl¯**; (b, e) DAPI; (c, f) merged images ([**2·2Cl¯**] = 2.5 × 10^{-6} M, λ > 420 nm, 220 mW/cm^2). The bars are 20 μm (white) and 30 μm (red), respectively.

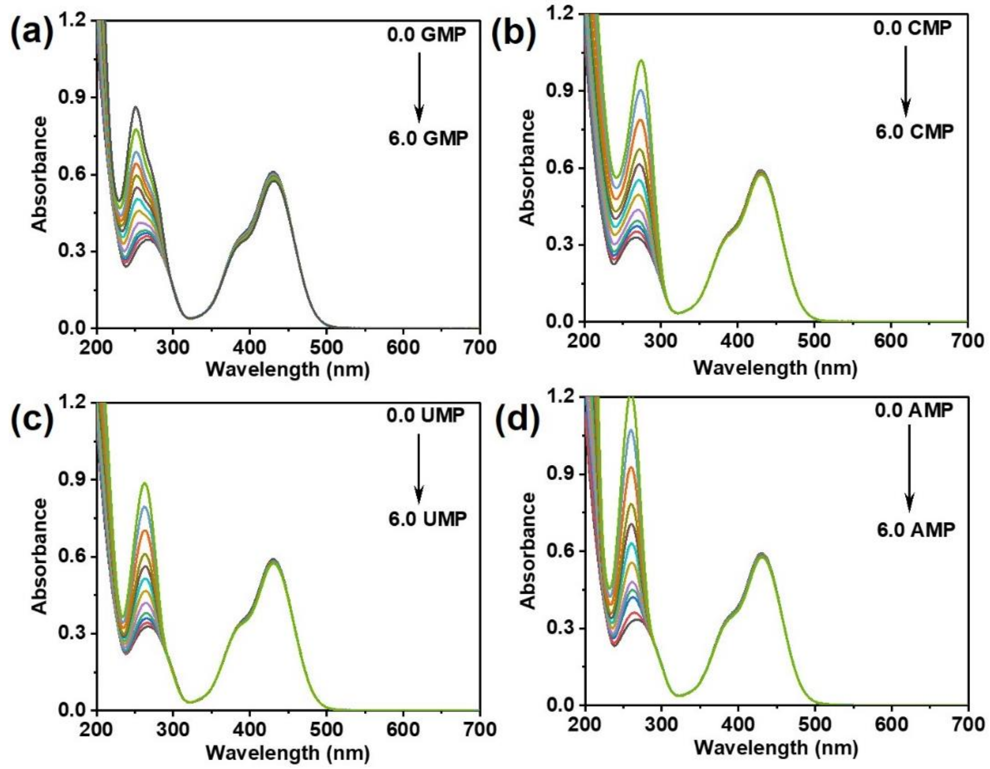

Figure S27. Uv-vis absorbance changes of **1·4Cl¯** upon addition of (a) guanosine monophosphate (GMP), (b) cytidine monophosphate (CMP), (c) uridine monophosphate (UMP), (d) adenosine monophosphate (AMP) ([**1·4Cl¯**] = 1 × 10^{-5} M, and [GMP] = [CMP] = [UMP] = [AMP] = 0–6 × 10^{-5} M).

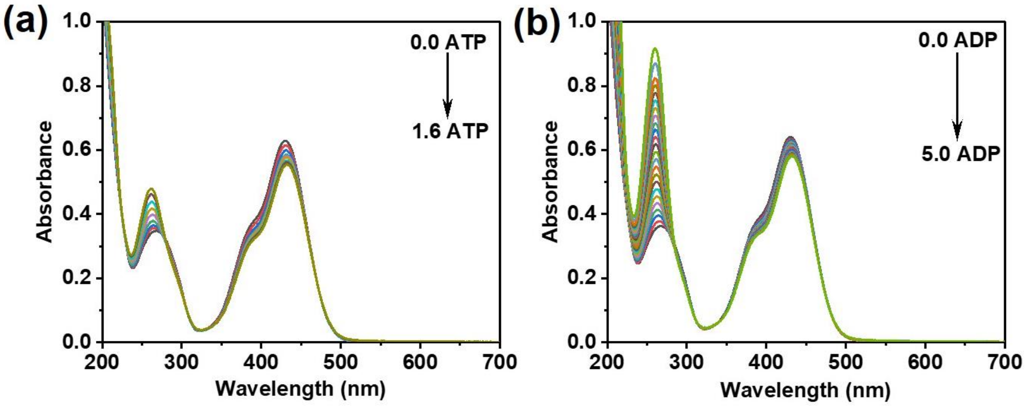

Figure S28. Uv-vis absorbance changes of **1·4Cl¯** upon addition of ATP (a), ADP (b). ([**1·4Cl¯**] = 1 × 10^{-5} M, [ATP] = 0-1.6 × 10^{-5} M, [ADP] = 0-5 × 10^{-5} M).

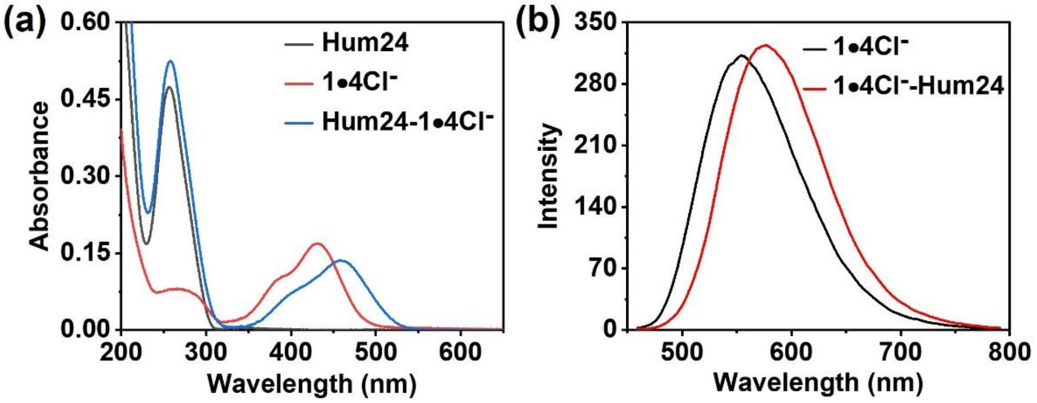

Figure S29. (a) Uv-vis absorption and (b) fluorescence emission spectra of free **1·4Cl¯**, free Hum4, and **1·4Cl¯** with Hum24 ([**1·4Cl¯**] = 2.5 × 10^{-6} M and [Hum24] = 1.5 × 10^{-6} M).

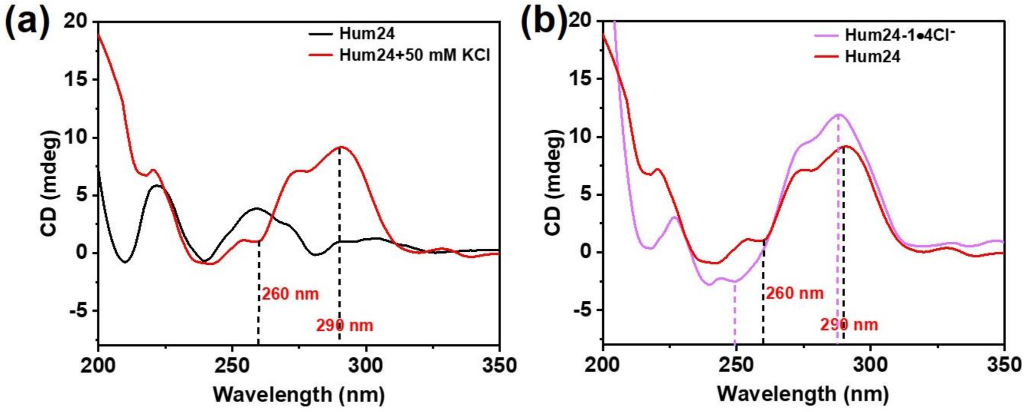

Figure S30. CD spectra of (a) Hum24 and G4 (Hum24 + 50 mM KCl), and (b) G4 and G4 + **1·4Cl¯** ([**1·4Cl¯**] = 2.5 × 10^{-6} M and [Hum24] = 1.5 × 10^{-6} M).

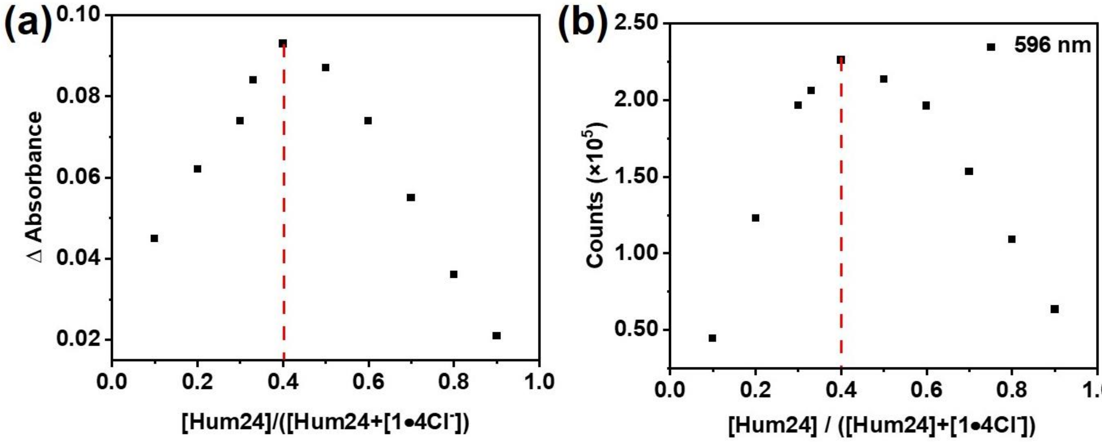

Figure S31. Job plots of Hum24 and **1·4Cl¯** obtained by (a) Uv-vis and (b) fluorescence emission spectroscopy (([**1·4Cl¯**] + [Hum24]) = 5 × 10^{-6} M).

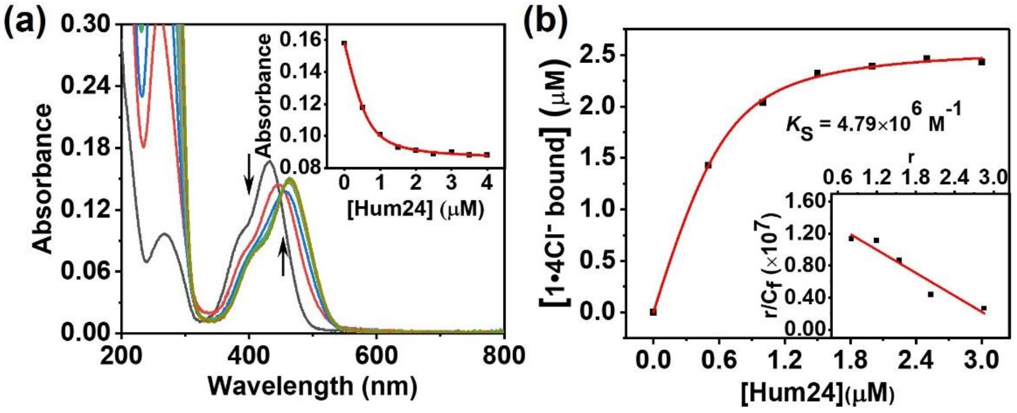

Figure S32. (a) Uv-vis titration spectra of **1·4Cl¯** upon addition of Hum24 (the concentration of mother solution is 1.0×10^{-5} M); Insert: Uv-vis absorbance changes of **1·4Cl¯** at 423 nm. (b) Fitting curve and Scatchard analysis (inset) for binding of **1·4Cl¯** with Hum24 ([**1·4Cl¯**] = 2.5×10^{-6} M and [Hum24] = 0-4 × 10^{-6} M).

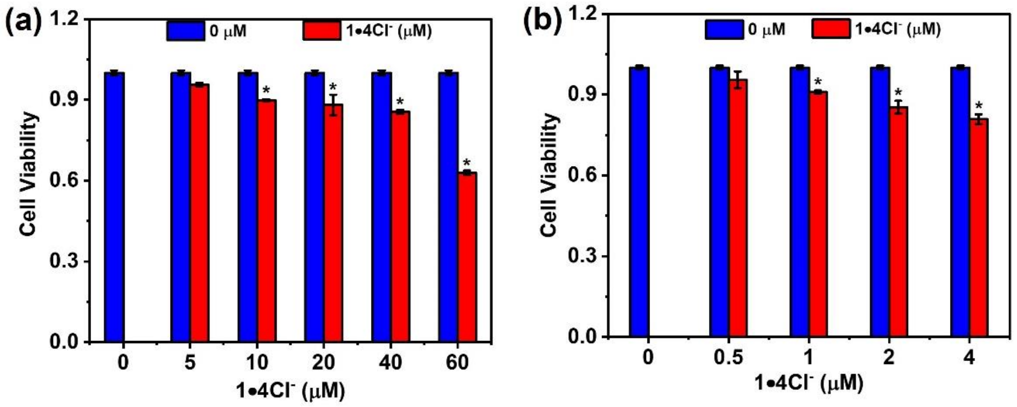

Figure S33. Cell viability at different concentrations of **1·4Cl¯** in 293T cells (a) without and (b) with light irradiation (λ > 420 nm, 220 mW/cm^2). Three independent experiments, with the data indicating mean ± SD. Asterisk * indicates a significant difference between the control group (0 μM **1·4Cl¯** without irradiation and with irradiation) and treated group (**1·4Cl¯** without irradiation and with irradiation) (p < 0.05).

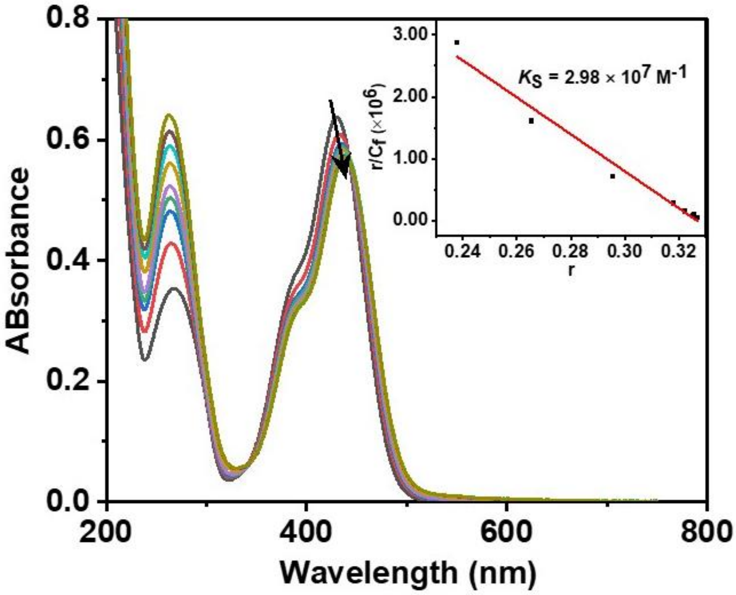

Figure S34. Uv-vis absorbance changes of **1·4Cl¯** upon addition of DNA and insert graph was the Scatchard plots ([**1·4Cl¯**] = 1 × 10^{-5} M and [DNA] = 0–1.5 × 10^{-2} mg/mL).

References

[S1] B. A. R. Peacocke, J. N. H. Skerrett, *Trans. Faraday Soc,* **1956**, *52*, 261-279.

[S2] J. D. Mcghee, P. H. Hippel, *J. Mol. Biol.* **1974**, *86*, 469-489.

[S3] M. Zhang, Q. Dai, H, Zheng, M. Chen, L. Dai, *Adv. Mater.* **2018**, *30*, 1705431.
